# Protein Consumption and Risk of CVD Among U.S. Adults: The Multi-Ethnic Study of Atherosclerosis (MESA)

**DOI:** 10.3390/nu16213773

**Published:** 2024-11-02

**Authors:** Ji Yun Tark, Ruosha Li, Bing Yu, Alexis C. Wood, Nikhil S. Padhye, Marcia C. de Oliveira Otto

**Affiliations:** 1 Department of Epidemiology, School of Public Health, The University of Texas Health Science Center at Houston, Houston, TX 77030, USA; ji.yun.tark@uth.tmc.edu (J.Y.T.); bing.yu@uth.tmc.edu (B.Y.); 2 Section of Epidemiology and Population Sciences, Department of Medicine, Baylor College of Medicine, Houston, TX 77030, USA; 3 Department of Biostatistics and Data Science, School of Public Health, The University of Texas Health Science Center at Houston, Houston, TX 77030, USA; ruosha.li@uth.tmc.edu; 4 Department of Pediatrics, USDA/ARS Children’s Nutrition Research Center, Baylor College of Medicine, Houston, TX 77030, USA; alexis.wood@bcm.edu; 5 Department of Research, School of Nursing, The University of Texas Health Science Center at Houston, Houston, TX 77030, USA; nikhil.s.padhye@uth.tmc.edu

**Keywords:** protein intake, diversity of protein sources, CVD

## Abstract

Background: Although some randomized trials have reported beneficial effects of protein intake on cardiometabolic risk factors, evidence from prospective studies have not supported a strong link between protein intake and cardiovascular disease (CVD) risk. It is also unclear whether diversity in protein intake plays a role in CVD risk. Objective: We investigated prospective associations of (1) protein intake, overall and by food source and (2) diversity of protein sources with risk of CVD, coronary heart disease (CHD), and stroke. Methods: In a multi-ethnic cohort of 5879 U.S. adults (45–84 years), who were free of CVD at baseline, protein intake was assessed at baseline (2000–2002) using a validated 120-item food frequency questionnaire. Two different aspects of protein diversity were assessed including count (number of protein food consumed at least once/week) and dissimilarity (diversity of the attributes of the protein sources consumed). Relationships with incident CVD outcomes through 2019 were assessed using Cox proportional hazards models adjusting for sociodemographic, lifestyle, and comorbidity factors. Results: During 83,430 person-years, 1045 CVD cases were identified, including 668 CHD and 332 stroke cases. In multivariable models, we found no significant associations between protein intake, overall and by food source, with incident CVD, CHD, or stroke. Protein count, but not protein dissimilarity, was weakly associated with CVD risk. We found no significant associations between diversity of consumption of animal or plant food source and CVD outcomes. Conclusions: Our findings suggest protein consumption may not significantly impact CVD risk in middle-aged adults.

## 1. Introduction

Cardiovascular disease (CVD) ranks as the foremost cause of mortality in the United States (US) [1]. Coronary heart disease (CHD) and stroke are the leading causes, responsible for approximately 60% of CVD-related deaths [2]. These conditions, along with hypertension, heart failure, and arterial diseases, are not only associated with a higher risk of mortality but also result in a significant economic burden. They cost the healthcare system about $251 billion annually and result in $156 billion in lost productivity in the US [2]. Projections indicate that CVD will affect 131 million individuals by 2035, nearly half of the current US population [3]. This underscores the urgent need for more effective interventions and policy measures.

Diet is the leading risk factor for cardiovascular health, with the potential to both improve risk factors and lower the incidence of CVD, as evidenced by findings from clinical trials [4,5,6,7,8] and observational studies [9,10,11,12,13]. The Dietary Guidelines for Americans recommends adhering to heart-healthy dietary patterns to improve cardiovascular health [14]. This includes a wide variety of protein sources including nuts and seeds, legumes, red meat, poultry, fish, and seafood. Despite its important role in healthy eating patterns, the impact of protein intake on cardiovascular disease (CVD) risk has not been fully established.

Proteins constitute almost 75% of most human tissues, aside from bone and adipose tissue [15]. Dietary proteins include nine essential amino acids which cannot be synthesized by humans in sufficient quantities. Consequently, these amino acids must be obtained through diet [15]. Randomized trials have shown modest benefits of protein intake on established cardiometabolic risk factors. For example, a meta-analysis of 54 randomized trials (duration range: 4–156 weeks) including 4344 participants (average age 46 years) showed that high protein diets (constituting 20–45% of total energy intake) were associated with reductions in systolic blood pressure (SBP) [standardized mean difference (95% CI): −0.12 (−0.21, −0.02)], total cholesterol [standardized mean difference (95% CI): −0.11 (−0.19, −0.02)], triacylglycerol [standardized mean difference (95% CI): −0.22 (−0.30, −0.14)], insulin [standardized mean difference (95% CI): −0.12 (−0.22, −0.03)], and fat mass [standardized mean difference (95% CI): −0.14 (−0.24, −0.04)], compared to lower protein diets (10–23% of energy intake) [16]. Despite the hypothesized benefits to cardiovascular health, evidence from prospective studies has not supported a strong link between protein intake and CVD events [15,17,18,19]. The absence of an association could partly relate to divergent effects of animal and plant-based protein sources on CVD risk. In addition, although dietary guidelines recommend consuming a variety of protein foods to ensure a balanced intake of essential nutrients—including amino acids—to optimize nourishment and reduce chronic disease risk [14], how protein diversity relates to cardiovascular health remains poorly understood.

To address these important knowledge gaps, we investigated the prospective associations of daily protein intake, both overall and by food source, as well as the diversity of protein sources, with incident CVD among participants in the Multi-Ethnic Study of Atherosclerosis.

## 2. Materials and Methods

### 2.1. Study Design and Population

The Multi-Ethnic Study of Atherosclerosis (MESA) is a prospective cohort study designed to investigate risk factors contributing to subclinical CVD within a racially and ethnically diverse population [20]. Briefly, from 2000 to 2002, the study recruited 6814 individuals (38% White, 28% Black/African American, 22% Hispanic, and 12% Chinese) aged between 45 and 84, free of CVD across six study sites: Columbia University in New York, Johns Hopkins University in Baltimore, Northwestern University in Chicago, the University of California in Los Angeles, University of Minnesota in Twin Cities, and Wake Forest University in Winston Salem. Each participant provided written informed consent.

### 2.2. Dietary Assessment

At baseline, MESA participants reported dietary intake for the past year using a 120-item self-administered food frequency questionnaire (FFQ) [21,22]. This block-type self-administered 120-item FFQ used in the MESA was developed based on the validated Block format [23] and modeled after the Insulin Resistance and Atherosclerosis Study (IRAS) FFQ [24]. The FFQ was modified by adding Chinese foods and beverages to accommodate Chinese Americans’ unique foods, such as stir-fried vegetables, spring rolls, dim sum, soya milk, and miso soup [24,25]. The validity of the parent FFQ was evaluated against eight 24 h recalls in Hispanics, African Americans, and non-Hispanic Whites [26], and the criterion validity of the modified MESA-specific FFQ was evaluated against plasma lipid concentrations within the MESA cohort [27]. For each food item on the questionnaire, participants noted the frequency of consumption and the serving sizes they typically consumed. The daily intake of nutrients for each FFQ item was calculated using the Nutrition Data System for Research (NDSR database; Nutrition Coordinating Center, University of Minnesota, Minneapolis, MN, USA) [28].

### 2.3. Estimation of Protein Intake and Diversity

The primary protein sources included in this study were seafood; meat, poultry, and eggs; beans, peas, and lentils; nuts, seeds, and soy products; and dairy foods. Protein intake, overall and by food source, was estimated by multiplying the reported amount (frequency 3 serving size) of food consumed by the protein content of that food (NDSR, Nutrition Coordinating Center, University of Minnesota, Minneapolis, MN, USA) [23,28]. Similarly, the protein density, i.e., the percentage of energy intake from total, animal, and plant protein, was estimated by dividing energy intake from each protein type by the total caloric intake (NDSR, Nutrition Coordinating Center, University of Minnesota, Minneapolis, MN, USA).

Based on previous work [29], we estimated two distinct aspects of protein diversity: (1) count, and (2) dissimilarity of food items consumed. Characterized as the number of food items consumed at least once a week, count is the most commonly used measure of diet diversity to date [30], reflecting the variety of protein food sources in one’s diet. To estimate the dissimilarity in protein sources, i.e., the diversity in types of protein foods consumed based on shared or unique attributes relevant to metabolic health [29], we used the Jaccard Distance [29,31], defined as $J_{Dist}=\frac{1}{n_{c}}\sum \frac{B_{x+}C_{y}}{A_{xy+}B_{x+}C_{y}}$, where $n_{c}$ = number of paired comparisons; $A_{xy}$ = number of food attributes that are related to CVD shared by protein food item *x* and *y*; $B_{x}$ = number of food attributes of *x*; and $C_{y}$ = number of food attributes of *y*. We selected 11 different food attributes based on likely evidence for effects on cardiometabolic health (Table S1). The count and dissimilarity measures were evaluated based on total protein intake, as well as separately for animal and plant foods.

### 2.4. Ascertainment of CVD

The primary outcomes in the study were total CVD, defined as myocardial infarction, resuscitated cardiac arrest, definite and probable angina, CHD death, stroke, stroke death, other atherosclerotic death, or other CVD death. CHD was defined as myocardial infarction, resuscitated cardiac arrest, CHD death, and angina, and stroke events comprised stroke and stroke death. Data on CVD incidence were collected through cohort examinations, follow-up calls, and the review of medical records and obituaries. Approximately 98% of reported hospitalized CVD events and 95% of outpatient diagnoses were achieved in MESA [32]. Reported diagnoses and related documents, including death certificates, autopsy reports, and medical records, were reviewed by a medical endpoints committee. For participants lost to follow-up, death confirmations were obtained by contacting family members. A standardized protocol was utilized to classify events and ascertain the dates of incidents based on the records available [22,33]. The present study includes 5900 participants who were free of CVD at baseline. We have excluded those with extreme daily energy intakes (<600 or >6000 kcal) [24] (*n* = 337) or incomplete dietary assessments (*n* = 577).

### 2.5. Assessment of Covariates

At baseline, detailed information on participants’ medical history, demographics, smoking habits, anthropometric data, blood pressure, and laboratory results were collected using standardized questionnaires and laboratory protocols [20]. Medication use was recorded by clinical staff who obtained all prescribed medications brought to the clinic visit by the participants [34]. Physical activity was assessed with a validated questionnaire designed to measure the duration and frequency of various physical activities [35]. The study considered numerous covariates, including sex (male and female), age, race/ethnicity (White, Black/African American, Hispanic, Chinese), study site, income (<$20,000, $20,000 to 49,900, and ≥$50,000), education level (<high school, high school, and >high school), physical activity (MET-min/week), alcohol intake (g/day), smoking status (former, current, and never), cigarette smoking (pack/years), body mass index (BMI) (kg/m^2^), dietary supplement use (yes/no), diabetes mellitus (yes/no), total energy intake (kcal/day), and the intakes of total fiber (g/day), saturated fatty acids (SFA) (%), trans-fat (%), monounsaturated fatty acids (MUFA) (%), PUFA (%), and vitamins E (IU/day). Additionally, potential mediators such as SBP (mmHg), diastolic blood pressure (DBP) (mmHg), triglycerides (mg/dL), high-density lipoproteins (HDL) (mg/dL), LDL (mg/dL), c-reactive protein (CRP) (mg/L), and interleukin-6 (IL-6) (pg/mL) levels were included. We used these variables measured at baseline.

### 2.6. Statistical Analysis

We performed a Spearman partial correlation analysis to assess the correlations between total protein intake, protein intake from animal and plant food sources, the diversity metrics in protein sources, and protein food groups, adjusting for age, sex, race/ethnicity, and energy intake. We evaluated the assumption underlying the proportional hazard model using Schoenfeld residuals and the significance test of time-dependent covariates included in Cox models and found no evidence of violation of the proportional hazard assumptions. In addition, we found no evidence of nonlinear associations between each protein metric and the outcomes of interest based on restricted cubic spline analysis [36]. We used Cox proportional hazard models to evaluate the associations of protein intake from total, animal, and plant food sources, as well as diversity of protein from total, animal, and plant food sources, with CVD incidence, with time at risk from the baseline exam until the first CVD event, death, or last follow-up in 2016–2018. Protein intake variables were assessed both as continuous variables and as categorical variables using quintiles. In addition, we assessed linear trends by assigning the median value in each quintile and evaluating this variable on a continuous scale [37].

To evaluate and minimize potential confounding factors, serial models were built adjusting for covariates based on biological interest, risk factors for CVD among adults, or associations with protein intake or CVD. Model 1 included age, sex, race/ethnicity, field center, and total energy intake. Model 2 included all the covariates from Model 1 plus highest education level, income, smoking status, pack-years of cigarette smoking, dietary supplement use, alcohol use, physical activity, prevalent diabetes, and BMI (kg/m^2^). Model 2 was the primary model. We included potential risk factors in Model 3a or Model 3b that could confound or mediate the association. Model 3a included all the covariates from Model 2 plus intakes of total dietary fiber, trans fats, SFA, PUFA, MUFA, and vitamin E. Model 3b included all the covariates from Model 2 plus SBP, DBP, triglycerides, HDL, LDL, CRP, and IL-6. For missing covariate data (<3% for most covariates except 35% missing for dietary supplement use), we applied a single imputation. This approach involved filling in missing entries with values estimated from a regression equation, which was adjusted for the covariates from Model 2 [38]. Previous research within MESA has shown that when missing data are minimal, single and multiple imputation methods yield similar results [39]. Additionally, we tested for multicollinearity between total protein intake and the covariates in Model 2 using generalized variation inflation factors (GVIF) [40]. There was no evidence of multicollinearity (GVIFs for all covariates < 2).

In order to assess the potential bias due to reverse causation [37], we conducted sensitivity analysis excluding individuals who developed CVD within the first two years of follow-up [41]. In addition, we compared results from statistical models with and without diabetes and BMI as covariates as these covariates could be both confounders and mediators for protein–CVD associations.

We assessed potential interactions based on sociodemographic factors by adding a multiplicative interaction term between each of the selected protein metrics and variables such as race/ethnicity (White, Black/African American, Hispanic, and Chinese), sex (male and female), education (high school graduate or less and some college and above), income (<$20,000, $20,000 to 49,900, and ≥$50,000), or age (<65 and ≥65 years) in Model 2.

We performed data analysis using SAS version 9.4 (SAS Institute, Cary, NC, USA), StataMP 16 (Stata Corp, College Station, TX, USA) or R Studio Version 1.3.959 (R Core Team, Vienna, Austria) with a two-sided *p*-value (*p* < 0.05), except for interaction analysis. The *p*-value for interaction analysis was adjusted using the False Discovery Rate (FDR) control procedure at a 0.05 significance (6 primary exposures × 5 interaction variables = 30 comparisons) [42].

## 3. Results

At baseline, the mean age ± standard deviation (SD) of 5879 participants was 62.3 years ± 10.3 (range 44–84 years), with 48% being male (Table S2). The majority of the participants were Whites (40%), followed by Blacks/African Americans (26%), Hispanics (22%), and Chinese (12%). The mean ± SD daily protein intake at baseline was 66.1 ± 14.0 g/day, with 42.4 ± 14.7 g/day from animal sources and 23.2 ± 6.9 g/day from plant sources. Protein intake from all sources accounted for nearly 16% of energy intake. The mean ± SD diversity of different protein food sources was 8.9 ± 4.1 for count (range: 0–31 food sources) and 0.68 ± 0.06 for dissimilarity (range: 0–1).

Participants consuming higher amounts of total protein tended to be younger, male, White, and less likely to be Chinese (Table 1). They were also more likely to have higher income levels, greater educational attainment, and be more physically active; additionally, they consumed more fruits and vegetables compared to those consuming lower amounts of total protein. In contrast, compared with participants with lower total protein intake, those with a higher total protein intake were more likely to have higher BMI (kg/m^2^) and be current smokers. The characteristics of participants with higher animal protein intake were similar to those observed in the group with higher total protein intake (Table S3). Participants in the higher quintile of plant protein intake were less likely to be White and more likely to be physically active and consume more fruits and vegetables; they were also less likely to smoke (Table S4).

### 3.1. Correlations Between Protein Exposures

Total protein intake (g/day) was strongly correlated with protein intake from animal sources (g/day) (r = 0.87) and also moderately correlated with protein intake from plant sources (r = 0.13), after adjusting for age, sex, race/ethnicity, and energy intake (Table 2). Consistently, total protein intake (g/day) showed a strong correlation with the percentage of energy from animal protein (r = 0.81), and moderate correlation with the percentage of energy from plant protein sources (r = 0.11) (Table S5).

When we evaluated correlations with protein diversity, total protein intake was positively correlated with protein count (r = 0.37), and inversely correlated with the dissimilarity of protein sources (r = −0.19) (Table 2).

When we evaluated correlations of protein intake by food source, protein from animal food sources (g/day) was negatively correlated with protein intake from plant sources (g/day) (r = −0.30), positively correlated with protein count (r = 0.33), and negatively correlated with dissimilarity (r = −0.24). On the other hand, protein intake from plant sources displayed moderate positive correlations with both the count (r = 0.12) and dissimilarity (r = 0.12).

**Figure 1:**
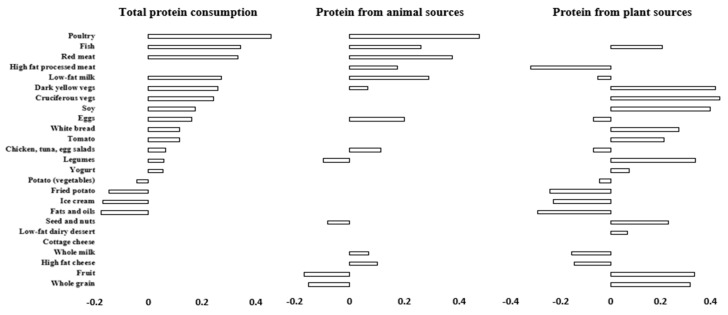
Spearman correlations between food groups and protein intake in 5879 multi-ethnic U.S. adults. Correlations were adjusted for age, sex, race/ethnicity, and energy intake. Only significant associations (*p*-value < 0.05) are shown. The bars represent correlation coefficients (ρ, rho).

When evaluating top correlations between major food groups and protein intake, total protein intake displayed weak to moderate positive correlations with poultry, red meat, fish, low-fat milk, dark yellow and cruciferous vegetables, soy, and eggs (r = 0.17 to 0.46), and moderate to weak negative correlation with fats and oils, ice cream, fried potato, and fruit (r = −0.16 to −0.03) (Figure 1). For protein from animal sources, the food groups that were correlated were similar to those in the total protein intake. Protein from plant sources was positively correlated with dark yellow and cruciferous vegetables, soy, legumes, fruit, and whole grain (r = 0.31 to 0.40) and inversely correlated with high-fat processed meat, fried potato, ice cream, fats and oils, and high-fat cheese (r = −0.30 to −0.19).

### 3.2. Associations Between Total Protein Intake, Protein Source Diversity, and Cardiovascular Disease Risk

During 83,430 person-years of follow-up between 2000 and 2019 [median (min, max) 17.5 (0.0,19.4) years], a total of 1045 new cases of CVD were identified, including 668 CHD and 332 stroke events. In multivariable models adjusting for energy intake, sociodemographic, lifestyle, and comorbidity factors, we found no statistically significant association between total protein intake and CVD risk (Table 3, Model 2). For example, HRs for each 20 g/day greater intake of total protein intake was 1.04 (95% CI 0.95–1.14) (Table 3, Model 2). Overall, consistent findings were observed for CHD or stroke as outcomes (Table 3, Model 2). Results were consistent across different age groups, genders, race-ethnicities, levels of education, and income (Table S17–S21). All findings were similar when evaluating the percent energy from total protein intake (Table S6, Model 2).

When we evaluated protein source diversity, the protein count, but not protein dissimilarity, was marginally associated with a higher CVD risk (HRs (95% CI); for each one additional count of total protein sources, the hazard ratio was 1.02 (1.00–1.04) (Table S11, Model 2). Associations were similar when we restricted the analysis to CHD outcomes (Table S11, Model 2). We found no association between the total protein count or dissimilarity and stroke risk (Tables S11 and S14, Model 2). Results remained consistent across different age groups, genders, and race-ethnicities.

### 3.3. Associations Between Animal Protein Intake and Diversity, and Cardiovascular Disease Risk

When we assessed protein from animal sources, we found no statistically significant associations with CVD, CHD, or stroke risk (Table S7, Model 2). For example, the HRs for each 20 g/day greater intake of protein from animal food sources were 1.04 (95% CI 0.96–1.13) (Table S7, Model 2). Similarly, we found no association between the diversity of animal protein sources (count or dissimilarity) and CVD outcomes (Tables S12 and S15, Model 2). All findings were consistent when evaluating the percent energy from animal protein intake (Table S8; Figures S1, S3 and S5). Results remained consistent across different age groups, genders, race-ethnicities, levels of education, and income (Tables S17–S21).

### 3.4. Associations Between Plant Protein Intake and Diversity, and Cardiovascular Disease Risk

When we assessed protein from plant foods, we found no statistically significant associations with CVD, CHD, or stroke risk (Table S9, Model 2). For example, the HRs for each 20 g/day greater intake of protein from plant food sources were 0.99 (95% CI 0.82–1.20) (Table S9, Model 2). In contrast, a higher percentage of energy from plant protein was associated with a lower risk of stroke, but not CVD or CHD [HR for each 5% of energy increment from plant protein was 0.62 (95% CI 0.41–0.93) (Table S10, Model 2)].

A higher plant protein count, but not dissimilarity, was marginally associated with higher CVD and CHD risk (Table S13, Model 2). For example, the HR for each additional plant protein food per day was 1.04 (95% CI 1.00–1.09) (Table S13, Model 2). In contrast, we found no association between plant protein diversity and stroke risk (Tables S13 and S16, Model 2). Results remained consistent across different age groups, genders, race-ethnicities, levels of education, and income (Tables S17–S21).

### 3.5. Sensitivity Analysis

Our findings remained materially unchanged after excluding 126 participants who developed CVD within the first two years of the follow-up period.

Upon removing adjustment for diabetes and BMI (kg/m^2^) from Model 2, higher intakes of total and animal protein intake were marginally associated with higher risks of CVD [HR (95% CI) for each 20 g increment in total and animal protein: 1.10 (1.00, 1.20) and 1.09 (1.00, 1.18), respectively] (Table 3 and Table S7, Model 2-BMI, diabetes).

Results were similar when we excluded individuals with self-reported kidney disease [Model 2 HR for each 20 g/day 1.00 (95% 1.00–1.01); HR for extreme quintiles 1.02 (0.94–1.09), *n* = 5735].

## 4. Discussion

In this large multi-ethnic cohort, we found no statistically significant associations between protein intake, overall and by food source, with incident CVD, CHD, or stroke. Protein count, but not protein dissimilarity, was weakly associated with CVD risk. We found no statistically significant associations between the diversity of consumption of animal or plant food source and CVD outcomes. These findings were similar among men and women and among Whites, Hispanics, Blacks, and Asians. Our findings build and expand on previous studies by evaluating associations with food-based protein intake overall, and by major food sources, and by utilizing two different metrics of protein diversity in a large diverse cohort of middle-aged U.S. adults. Our findings have important implications, suggesting that protein food sources can be consumed without significantly increasing the risk of CVD among middle-aged U.S. adults.

Our results are consistent with findings from previous cohort studies evaluating associations with CVD in middle-aged and older adults. For example, the Prospective Urban Rural Epidemiology study, which included 135,335 individuals with 4784 CVD incidence from Canada, Sweden, Argentina, Brazil, India, and other 13 countries, showed no statistically significant associations between total protein intake and CVD risk [HR (95% CI) for extreme quintiles of protein intake: 0.96 (0.84, 1.10) after a median follow-up of 7.4 years] [18]. Recent findings from the European Prospective Investigation into Cancer and Nutrition case–cohort study, including 16,244 CVD cases and 15,141 sub-cohort from European countries, showed neither plant nor animal protein intake was associated with CVD risk [43]. A meta-analysis of five prospective cohort studies in US, Japan, and the Netherlands, which included 245,222 participants and 14,704 CVD deaths, found no statistically significant association between total or animal protein intake and the risk of CVD mortality [44]. A meta-analysis including 10 prospective cohort studies in US, Spain, Japan, and other 19 countries, which included 427,005 participants and 15,518 CVD deaths, reported an inverse association between plant protein, but not total or animal protein intake, and risk of CVD mortality [pooled HR (95% CI): 0.88 (0.80, 0.96)] [45].

Previous observational studies support the cardiovascular benefits of major protein sources such as legumes, nuts, low-fat dairy, and fish [46,47,48,49,50]. However, a growing body of evidence indicates potential health risks associated with highly processed foods, including some plant-based meats and dairy substitutes [51,52]. For instance, a recent UK Biobank cohort study found that minimally processed plant-based foods like nuts, seeds, and legumes were linked to a reduced CVD risk [adjusted HR (95% CI): 0.93 (0.91–0.95)], while highly processed plant-based foods—such as margarine, meat alternatives, and salty snacks—were associated with an increased CVD risk [adjusted HR (95% CI): 1.05 (1.03–1.07)] over a median follow-up of 9 years among 126,842 participants [52]. In addition to providing essential amino acids, ultra-processed plant protein sources often contain high levels of sodium, trans fats, added sugars, and food additives, which may adversely impact CVD risk factors such as hypertension, obesity, and type 2 diabetes [53,54,55,56,57,58,59,60]. The differences in food processing, nutrients, and other food components could partially explain the lack of an association with protein intake. Therefore, more research is needed to further understand the cardiovascular implications of food processing on health effects of protein food sources.

It is also plausible that protein quality plays a crucial role in the relationship between protein intake and cardiovascular health. Protein quality refers to the ability of a dietary protein source to deliver essential amino acids necessary for the body’s growth, maintenance, and repair [61]. High-quality proteins, being readily absorbed, may have a different impact on cardiovascular health than lower-quality proteins. Further research is warranted to explore these distinctions. Importantly, our study does not question the benefits of consuming protein-rich foods, as recommended by the Dietary Guidelines for Americans [14]. Rather, our findings suggest that including protein food sources as part of a balanced dietary pattern may not lead to an increased CVD risk among middle-aged adults.

Our study has several strengths. Information on diet and covariates were prospectively collected, establishing temporality and minimizing concerns with reverse causality. Our analysis using data from the MESA study allowed the study of a diverse U.S cohort with a wide range of dietary behaviors and susceptibility to CVD outcomes, increasing confidence in the validity and generalizability of the results. We adjusted for multiple covariates, thus minimizing confounding. The use of both protein intake and diversity metrics allowed a comprehensive assessment of the relationships of interest.

Our study has potential limitations. The use of self-reported dietary measures may lead to potential measurement error in protein metrics that could lead to attenuation in measures of association [62]. In addition, as previously indicated, the use of FFQ to assess diversity may have limited our ability to fully assess the entire range of protein foods consumed, thereby limiting accuracy in diversity metrics, particularly when assessing diversity of animal and plant food sources [29]. Therefore, our results may have underestimated true associations. On the other hand, FFQs are well-suited for assessing long-term dietary intake, which are more relevant to chronic disease risk [37,63]. Lastly, although we were able to control for various major risk factors, the potential for unmeasured or poorly measured confounding cannot be dismissed.

## 5. Conclusions

In summary, in this large study of multi-ethnic, middle-aged U.S. adults, we found no strong evidence linking total protein intake—whether overall, animal-based, plant-based, or protein diversity—with CVD risk. Public health recommendations should continue to encourage a variety of minimally processed protein foods from both animal and plant sources, such as seafood, nuts, and legumes, to support dietary needs and overall health.

## Figures and Tables

**Table 1:** Characteristics of MESA participants according to quintiles of total protein intake (*n* = 5879) ^1^.

	Quintiles of Total Protein Intake (g) Measured at Exam 1				
Characteristic	Q1	Q2	Q3	Q4	Q5
*n*	1172	1177	1178	1176	1176
Mean (min, max)	32.0 (11.1, 39.9)	46.3 (39.9, 53.1)	60.1 (53.1, 67.2)	76.2 (67.2, 87.9)	116.0 (87.9, 261.6)
**Demographic factors**					
Age, y	64.3 ± 10.4	63.4 ± 10.4	62.1 ± 9.9	61.7 ± 10.0	59.8 ± 10.2
Male, %	34.6	41.7	47.7	53.1	60.8
Race/ethnicity, %					
White	32.3	37.8	45.3	45.8	40.1
Black/African American	28.6	25.7	24.5	23.6	27.3
Hispanic	21.0	20.7	18.9	23.4	26.0
Chinese	18.2	21.1	11.3	7.3	6.6
**Socioeconomic factors**					
Income, *%*					
<$20,000	29.1	25.7	19.5	20.0	21.7
$20,000–$49,999	39.3	37.6	35.6	36.9	37.0
≥$50,000	31.6	36.6	44.9	43.1	41.3
Highest education level, %					
<High school	21.9	19.6	14.2	16.9	17.1
High school	20.2	19.7	17.1	15.8	16.1
>High school	57.9	60.7	68.8	67.3	66.8
**Lifestyle and comorbidity factors**					
Current smokers, *%*	10.9	10.3	13.2	11.8	16.2
Cigarette pack-years	8.9 ± 16.7	11.5 ± 21.1	11.7 ± 20.5	12.1 ± 22.2	12.5 ± 22.5
Moderate and vigorous physical activity, *MET-min/week*	4907.9 ± 5057.7	5289.8 ± 5157.9	5462.3 ± 5482.9	6294.6 ± 6848.6	6705.6 ± 6615.0
Dietary supplement use, %	92.9	90.4	88.0	87.6	88.0
Prevalent diabetes, %	12.6	11.1	11.4	13.9	12.6
BMI, kg/m^2^	27.5 ± 5.5	27.8 ± 5.2	28.1 ± 5.4	28.6 ± 5.4	29.3 ± 5.4
**Dietary factors**					
Energy intake, kcal/d	938.6 ± 232.2	1247.4 ± 266.9	1559.4 ± 310.1	1935.1 ± 382.4	2793.5 ± 771.3
Alcohol intake, g/d	3.1 ± 9.4	4.3 ± 9.5	5.5 ± 12.6	6.5 ± 13.8	7.6 ± 17.8
Fruit intake, servings/day	1.7 ± 1.3	1.8 ± 1.4	2.1 ± 1.7	2.2 ± 1.7	2.4 ± 1.9
Vegetable ^2^ intake, servings/day	1.7 ± 1.0	2.1 ± 1.2	2.5 ± 1.3	2.7 ± 1.6	3.6 ± 2.0
Total fiber intake, g/d	12.4 ± 4.7	15.5 ± 5.5	18.8 ± 6.8	22.0 ± 8.0	29.5 ± 11.5
Saturated fat intake, *% of energy*	8.8 ± 3.1	9.6 ± 3.0	10.0 ± 3.1	10.6 ± 3.0	11.3 ± 2.9
Polyunsaturated fat intake, *% of energy*	5.9 ± 2.0	6.0 ± 1.7	6.0 ± 1.6	6.0 ± 1.7	6.1 ± 1.7
Monounsaturated fat intake, *% of energy*	11.0 ± 2.9	11.5 ± 2.7	11.9 ± 2.8	12.1 ± 2.7	12.6 ± 2.6
* trans* fat intake, *% of energy*	0.8 ± 0.3	0.8 ± 0.3	0.8 ± 0.3	0.8 ± 0.3	0.9 ± 0.3
Vit E intake, IU/d	5.2 ± 2.8	6.7 ± 3.0	8.2 ± 3.2	10.0 ± 3.8	14.2 ± 6.5
Protein intake ^3^, g/d					
Animal protein intake	37.0 ± 7.7	39.3 ± 9.8	41.0 ± 11.9	43.5 ± 14.6	51.2 ± 21.2
Plant protein intake	22.5 ± 3.8	23.1 ± 4.7	23.6 ± 6.0	23.3 ± 7.6	23.7 ± 10.3
Protein intake, *% of energy*					
Total protein	14.1 ± 3.0	15.4 ± 3.0	16.0 ± 3.0	16.3 ± 3.0	17.0 ± 3.0
Animal protein	8.0 ± 2.9	9.5 ± 3.1	10.1 ± 3.1	10.7 ± 3.3	11.6 ± 3.2
Plant protein	5.9 ± 1.6	5.8 ± 1.5	5.7 ± 1.6	5.4 ± 1.6	5.3 ± 1.5
Diversity of total protein foods					
Count	5.8 ± 2.8	7.5 ± 3.0	8.7 ± 3.2	10.0 ± 3.3	12.7 ± 4.3
Dissimilarity	0.68 ± 0.09	0.68 ± 0.07	0.68 ± 0.06	0.68 ± 0.05	0.68 ± 0.04

^1^ Values are percent or mean ± SD. MET, metabolic equivalent; BMI, body mass index. ^2^ Includes green leafy vegetables, cruciferous vegetables, dark yellow vegetables, other vegetables, and tomato food group. ^3^ Energy-adjusted.

**Table 2:** Spearman partial correlations among protein exposures in 5823 participants in MESA.

	Protein Intake from Animal Sources (g/Day)	Protein Intake from Plant Sources (g/Day)	Count of Total Protein Intake	Dissimilarity of Total Protein Sources
Total protein intake (g/day)	0.87	0.13	0.37	−0.19
Protein intake from animal sources (g/day)		−0.30	0.33	−0.24
Protein intake from plant sources (g/day)			0.12	0.12
Count of total protein sources				

Correlations were adjusted for age, sex, race/ethnicity, and energy intake. Only statistically significant coefficients are shown.

**Table 3:** Hazard ratios (95% CIs) of incident CVD, CHD, and stroke by quintile and continuous total protein intake in MESA (*n* = 5879).

				HR (95% CI)			
	Q1	Q2	Q3	Q4	Q5	$P_{trend}$	For 20 g/Day Increment
Total Protein Intake (Grams/Day)							
**CVD**							
Mean (min, max)	32.0 (11.1, 39.9)	46.3 (39.9, 53.1)	60.1 (53.1, 67.2)	76.2 (67.2, 87.9)	116.0 (87.9, 261.6)		
Cases	206	211	224	205	199		
Model 1	Reference	1.00 (0.82, 1.22)	1.14 (0.92, 1.41)	1.08 (0.84, 1.38)	1.16 (0.83, 1.61)	0.36	1.10 (1.00, 1.20)
Model 2	Reference	1.01 (0.83, 1.24)	1.12 (0.90, 1.38)	1.03 (0.80, 1.32)	1.06 (0.76, 1.48)	0.76	1.04 (0.95, 1.14)
Model 2-BMI, diabetes	Reference	1.02 (0.84, 1.25)	1.17 (0.95, 1.45)	1.11 (0.87, 1.43)	1.18 (0.84, 1.65)	0.32	1.10 (1.00, 1.20)
Model 3a	Reference	1.02 (0.84, 1.25)	1.12 (0.90, 1.40)	1.04 (0.81, 1.34)	1.07 (0.75, 1.51)	0.75	1.05 (0.95, 1.16)
Model 3b	Reference	1.04 (0.85, 1.26)	1.14 (0.92, 1.41)	1.03 (0.81, 1.32)	1.11 (0.79, 1.55)	0.61	1.06 (0.97, 1.16)
**CHD**							
Mean (min, max)	32.0 (11.1, 39.9)	46.3 (39.9, 53.1)	60.1 (53.1, 67.2)	76.2 (67.2, 87.9)	116.0 (87.9, 261.6)		
Cases	133	123	145	140	127		
Model 1	Reference	0.87 (0.68, 1.12)	1.04 (0.80, 1.35)	1.00 (0.74, 1.36)	0.95 (0.63, 1.43)	0.96	1.07 (0.96, 1.19)
Model 2	Reference	0.87 (0.67, 1.12)	1.00 (0.77, 1.30)	0.95 (0.70, 1.28)	0.86 (0.57, 1.30)	0.63	1.01 (0.90, 1.13)
Model 2-BMI, diabetes	Reference	0.88 (0.68, 1.13)	1.06 (0.81, 1.38)	1.03 (0.76, 1.40)	0.96 (0.63, 1.46)	0.91	1.07 (0.96, 1.20)
Model 3a	Reference	0.87 (0.67, 1.12)	0.99 (0.75, 1.30)	0.93 (0.68, 1.28)	0.84 (0.54, 1.30)	0.57	1.01 (0.89, 1.14)
Model 3b	Reference	0.89 (0.69, 1.14)	1.03 (0.79, 1.34)	0.95 (0.70, 1.28)	0.89 (0.59, 1.36)	0.74	1.02 (0.91, 1.15)
**Stroke**							
Mean (min, max)	32.0 (11.1, 39.9)	46.3 (39.9, 53.1)	60.1 (53.1, 67.2)	76.2 (67.2, 87.9)	116.0 (87.9, 261.6)		
Cases	70	77	72	56	57		
Model 1	Reference	1.17 (0.84, 1.64)	1.27 (0.87, 1.85)	1.07 (0.68, 1.69)	1.35 (0.74, 2.47)	0.46	1.13 (0.96, 1.33)
Model 2	Reference	1.17 (0.84, 1.64)	1.24 (0.85, 1.81)	1.01 (0.65, 1.60)	1.21 (0.66, 2.23)	0.73	1.07 (0.91, 1.27)
Model 2-BMI, diabetes	Reference	1.19 (0.85, 1.67)	1.29 (0.88, 1.88)	1.09 (0.69, 1.71)	1.33 (0.72, 2.44)	0.51	1.12 (0.95, 1.33)
Model 3a	Reference	1.18 (0.84, 1.66)	1.26 (0.86, 1.85)	1.03 (0.65, 1.64)	1.21 (0.64, 2.26)	0.75	1.07 (0.89, 1.28)
Model 3b	Reference	1.21 (0.87, 1.70)	1.28 (0.87, 1.86)	1.03 (0.66, 1.62)	1.27 (0.69, 2.35)	0.64	1.10 (0.93, 1.30)

HR indicates hazard ratio; CI, confidence interval; Q1, first quintile; Q2, second quintile; Q3, third quintile; Q4, fourth quintile; Q5, fifth quintile; CVD, cardiovascular disease; CHD, coronary heart disease; BMI, body mass index. HRs were obtained for quintiles of protein intake, with Q1 as the reference, and continuously for each 20 g/day increment in protein intake in relation to CVD risk. Linear trends were assessed by assigning the median value in each quintile and evaluating it on a continuous scale. Model 1 is adjusted for age (years), sex, race/ethnicity (White, Chinese, Black, Hispanic), field center, and total energy intake (kcal); Model 2 is adjusted for all variables in Model 1 plus education (<high school, high school, >high school), income (<$20,000, $20,000–$49,999, >$49,999), smoking status (current, former, never) and pack-years of cigarette smoking, dietary supplement use one per week or more (yes/no), alcohol use (g/day), physical activity (moderate and vigorous physical activity total, metabolic equivalents per min/week), prevalent diabetes (yes/no), and body max index (kg/m^2^); Model 2-BMI, diabetes is adjusted for all variables in Model 2 excluding BMI (kg/m^2^) and prevalent diabetes; Model 3a is adjusted for all variables in Model 2 plus intakes of total dietary fiber (g/day), trans fats (% of energy), saturated fatty acids (% of energy), poly-unsaturated fatty acids (% of energy), monounsaturated fatty acids (% of energy), and vitamin E (IU/day); Model 3b is adjusted for all variables in Model 2 plus systolic blood pressure (mmHg), diastolic blood pressure (mmHg), triglycerides (mg/dL), HDL cholesterol (mg/dL), LDL cholesterol (mg/dL), C-reactive protein (mg/L), and interleukin-6 (pg/mL).

## Data Availability

The data used in this study were obtained from the Multi-Ethnic Study of Atherosclerosis (MESA). MESA data are accessible to qualified researchers through application to the MESA Coordinating Center or through the NIH data repository.

## Supplementary Materials

The following supporting information can be downloaded at: https://www.mdpi.com/article/10.3390/nu16213773/s1, Table S1: Attributes of dissimilarity score; Table S2: Baseline characteristics of MESA participants free of CVD (*n* = 5879); Table S3: Characteristics of MESA participants according to quintiles of animal protein intake (*n* = 5879); Table S4: Characteristics of MESA participants according to quintiles of plant protein intake (*n* = 5879): Table S5: Spearman partial correlations among protein exposures in 5879 participants in MESA; Table S6: Hazard ratios (95% CIs) of incident CVD, CHD, and stroke by quintile and continuous animal protein in MESA: Table S7: Hazard ratios (95% CIs) of incident CVD, CHD, and stroke by quintile and continuous animal protein in MESA; Table S8: Hazard ratios (95% CIs) of incident CVD, CHD, and stroke by quintile and continuous percentage of energy intake from animal protein in MESA; Table S9: Hazard ratios (95% CIs) of incident CVD, CHD, and stroke by quintile and continuous plant protein intake in MESA; Table S10: Hazard ratios (95% CIs) of incident CVD, CHD, and stroke by quintile and continuous count of total protein in MESA: Table S11: Hazard ratios (95% CIs) of incident CVD, CHD, and stroke by quintile and continuous count of total protein sources in MESA; Table S12: Hazard ratios (95% CIs) of incident CVD, CHD, and stroke by quintile and continuous count of plant protein sources in MESA; Table S13: Hazard ratios (95% CIs) of incident CVD, CHD, and stroke by quintile and continuous count of total protein sources in MESA; Table S14: Hazard ratios (95% CIs) of incident CVD, CHD, and stroke by quintile and continuous dissimilarity of total protein sources in MESA; Table S15: Hazard ratios (95% CIs) of incident CVD, CHD, and stroke by quintile and continuous dissimilarity of plant protein sources in MESA; Table S16: Hazard ratios (95% CIs) of incident CVD, CHD, and stroke by quintile and continuous dissimilarity of plant protein sources in MESA; Table S17: Hazard ratios (95% CIs) of incident CVD by quintile and continuous protein intake measures stratified by race/ethnicity in MESA (*n* = 5879); Table S18: Hazard ratios (95% CIs) of incident hypertension by quintile and continuous protein intake measures stratified by sex in MESA (*n* = 5879); Table S19: Hazard ratios (95% CIs) of incident hypertension by quintile and continuous protein intake measures stratified by age in MESA (*n* = 5879); Table S20: Hazard ratios (95% CIs) of incident hypertension by quintile and continuous protein intake measures stratified by education in MESA (*n* = 5879); Table S21: Hazard ratios (95% CIs) of incident hypertension by quintile and continuous protein intake measures stratified by income in MESA (*n* = 5879); Figure S1: Multivariable-adjusted hazard ratios (HRs) of incident CVD according to total, animal, and plant protein intake (g/day and % energy), evaluated using restricted cubic splines; Figure S2: Multivariable-adjusted hazard ratios (HRs) of incident CVD according to diversity of protein sources, evaluated using restricted cubic splines. Figure S3: Multivariable-adjusted hazard ratios (HRs) of incident CHD according to total, animal, and plant protein intake (g/day and % energy), evaluated using restricted cubic splines; Figure S4: Multivariable-adjusted hazard ratios (HRs) of incident CHD according to diversity of protein sources, evaluated using restricted cubic splines; Figure S5: Multivariable-adjusted hazard ratios (HRs) of incident stroke according to total, animal, and plant protein intake (g/day and % energy), evaluated using restricted cubic splines; Figure S6: Multivariable-adjusted hazard ratios (HRs) of incident stroke according to diversity of protein sources, evaluated using restricted cubic splines. Reference [64] are cited in the Supplementary Materials.
