# Supplementary material for: Protein Consumption and Risk of CVD Among U.S. Adults: The Multi-Ethnic Study of Atherosclerosis (MESA)
Source: Nutrients. 2024 Nov 2;16(21):3773. doi: 10.3390/nu16213773 (PMC11548220; doi:10.3390/nu16213773)
Supplement: Supplementary file 1 [file nutrients-16-03773-s001.zip › nutrients-3251123-supplementary.pdf]

Table S1. Attributes of Dissimilarity Score

Attribute	Categories	Criterion
Food type	Animal<br>Plant	Main protein source
Food processing	Highly processed<br>Moderately processed<br>Minimally processed	Highly, Moderately, or Minimally Processed
Food structure	Liquid<br>Solid	
Fermentation	Yes<br>No	
Fiber content	High<br>Moderate<br>Low	tertiles of total fiber content per 100g of food
Total PUFA content	High<br>Moderate<br>Low	tertiles of PUFA content per 100g of food
EPA+DHA content	High	top tertile of EPA+DHA per 100g of food
Glycemic load (GL)	High	top tertile of GL
Antioxidants content	High<br>Moderate<br>Low	tertiles of antioxidant scores of food items based on nutrient content (vitamins A, C and E; β-, γ- and δ-tocopherol; β and α- carotene, lycopene and lutein[1])
Total trans-fat (TFA) content	High	>0.5g TFA per 100g of food
Sodium content	High<br>Moderate<br>Low	tertiles of sodium content per 100g of food

Adapted from de Oliveira Otto, M.C., et al.[2]

Table S2. Baseline characteristics of MESA participants free of CVD (*n*=5,879)[1]

Characteristic	
**Demographic factors**	
Age, *y*	62.3 ± 10.3
Men, *%*	47.6
Race/ethnicity, *%*	
White	40.2
Black/African American	25.9
Hispanic	22.1
Chinese	11.8
**Socioeconomic factors**	
Income, *%*	
<$20,000	23.2
$20,000 to $49,999	37.3
≥$50,000	39.5
Highest education level, *%*	
< High school	18.0
High school	17.8
> High school	64.3
**Lifestyle factors**	
Smoking status, *%*	
Never	50.4
Former	37.1
Current	12.5
Cigarette pack-years	11.3 ± 20.7
Moderate and vigorous physical activity, *MET-min/wk*	5732.5 ± 5916.4
Dietary supplement use, *%*	89.4
**Comorbidities**	
Prevalent diabetes, *%*	12.3
BMI, kg/m^2	28.3 ± 5.4
**Dietary factors**	
Energy intake, *kcal/d*	1695.2 ± 776.7
Alcohol intake, *g/d*	5.4 ± 13.1
Vit E intake, *IU/d*	8.8 ± 5.2
Total fiber intake, *g/d*	19.6 ± 9.7
Saturated fat intake, *% of energy*	10.1 ± 3.1
Polyunsaturated fat intake, *% of energy*	6.0 ± 1.7
Monounsaturated fat intake, *% of energy*	11.8 ± 2.8
trans fat intake, *% of energy*	0.8 ± 0.3
Fruit intake, *servings/day*	2.03 ± 1.65
Vegetable[2] intake, *servings/day*	2.50 ± 1.58
Protein intake[3], *g/d*	
Total protein	66.1 ± 14.0
Animal protein	42.4 ± 14.7
Plant protein	23.2 ± 6.9
Protein intake, *% of energy*	
Total protein	15.7 ± 3.2
Animal protein	10.0 ± 3.3
Plant protein	5.6 ± 1.6
Diversity of total protein foods	
Count	8.9 ± 4.1
Dissimilarity	0.68 ± 0.06

[1] Values are percent or mean ± SD. MET, metabolic equivalent; BMI, body mass index. [2] Includes green leafy vegetables, cruciferous vegetables, dark yellow vegetables, other vegetables, and tomato food group. [3] Energy-adjusted

Table S3. Characteristics of MESA participants according to quintiles of animal protein intake (n=5,879)[1]

	Quintiles of animal protein intake (g) measured at Exam 1				
Characteristic	Q1	Q2	Q3	Q4	Q5
n	1173	1176	1176	1178	1176
Mean (min, max)	16.6 (0.2, 22.6)	27.4 (22.6, 32.2)	37.4 (32.2, 43.1)	49.9 (43.1, 58.5)	80.4 (58.5, 210.0)
**Demographic factors**					
Age, y	64.5 ± 10.4	63.5 ± 10.3	62.2 ± 10.0	61.4 ± 10.2	59.7 ± 21.7
Male, *%*	38.1	43.5	47.1	51.4	57.8
Race/ethnicity, *%*					
White	33.3	38.1	42.7	46.0	41.1
Black/African American	26.6	25.9	25.4	23.5	28.3
Hispanic	21.0	20.0	21.9	23.1	24.3
Chinese	19.1	16.1	10.0	7.4	6.3
**Socioeconomic factors**					
Income, *%*					
< $20,000	27.3	26.7	21.5	20.2	20.3
$20,000-$49,999	40.2	36.6	34.9	36.8	38.0
≥ $50,000	32.5	36.7	43.6	43.0	41.7
Highest education level, *%*					
< High school	21.8	18.5	17.2	15.9	16.3
High school	18.8	19.6	17.4	17.5	15.5
> High school	59.3	61.8	65.4	66.6	68.2
**Lifestyle and comorbidity factors**					
Current smokers, *%*	9.7	9.5	13.2	13.8	16.2
Cigarette pack-years	8.3 ± 16.2	11.7 ± 20.8	11.7 ± 21.6	12.6 ± 22.5	12.4 ± 21.7
Moderate and vigorous physical activity, *MET-min/wk*	5203.4 ± 5313.4	5164.1 ± 5215.6	5624.4 ± 5754.4	6078.0 ± 5891.6	6590.4 ± 7098.6
Dietary supplement use, *%*	94.0	89.4	87.9	87.1	88.4
Prevalent diabetes, *%*	11.8	10.8	13.0	12.5	13.5
BMI, *kg/m^2*	27.2 ± 5.1	27.7 ± 5.3	28.3 ± 5.5	28.6 ± 5.2	29.5 ± 5.5
**Dietary factors**					
Energy intake, *kcal/d*	1039.2 ± 340.7	1280.4 ± 363.7	1569.0 ± 443.0	1916.2 ± 517.3	2669.0 ± 830.5
Alcohol intake, *g/d*	3.5 ± 10.1	4.5 ± 10.1	5.4 ± 11.7	6.0 ± 13.3	7.7 ± 18.1
Fruit intake, *servings/day*	1.9 ± 1.6	1.9 ± 1.6	2.0 ± 1.6	2.1 ± 1.6	2.2 ± 1.7
Vegetable[2] intake, *servings/day*	2.0 ± 1.5	2.2 ± 1.3	2.4 ± 1.4	2.6 ± 1.5	3.3 ± 1.9
Total fiber intake, *g/d*	15.3 ± 7.6	16.5 ± 7.4	18.9 ± 8.4	21.2 ± 8.8	26.3 ± 11.5
Saturated fat intake, *% of energy*	8.1 ± 2.8	9.4 ± 2.9	10.2 ± 2.9	10.8 ± 2.8	11.8 ± 2.9
Polyunsaturated fat intake, *% of energy*	6.1 ± 2.1	5.9 ± 1.7	6.0 ± 1.7	6.1 ± 1.6	6.0 ± 1.5
Monounsaturated fat intake, *% of energy*	10.6 ± 2.8	11.3 ± 2.7	12.0 ± 2.7	12.4 ± 2.6	12.9 ± 2.6
trans fat intake, *% of energy*	0.7 ± 0.3	0.8 ± 0.3	0.8 ± 0.3	0.9 ± 0.3	0.9 ± 0.3
Vit E intake, *IU/d*	5.9 ± 3.4	6.8 ± 3.4	8.2 ± 3.8	10.0 ± 4.3	13.3 ± 6.5
Protein intake[3], *g/d*					
Total protein intake	58.4 ± 8.3	62.3 ± 9.0	64.7 ± 11.0	67.6 ± 12.8	77.7 ± 18.2
Plant protein intake	24.8 ± 5.9	24.0 ± 5.6	23.6 ± 6.0	22.7 ± 6.7	21.2 ± 9.1
Protein intake, *% of energy*					
Total protein	13.5 ± 2.7	15.3 ± 2.8	15.9 ± 2.9	16.5 ± 2.9	17.5 ± 3.1
Animal protein	6.9 ± 2.4	9.2 ± 2.5	10.2 ± 2.8	11.1 ± 2.8	12.6 ± 3.2
Plant protein	6.5 ± 1.8	6.0 ± 1.6	5.6 ± 1.4	5.2 ± 1.3	4.8 ± 1.3

Diversity of total protein foods					
Count	5.7 ± 2.8	7.6 ± 3.1	8.8 ± 3.2	10.0 ± 3.4	12.5 ± 4.4
Dissimilarity	0.69 ± 0.09	0.68 ± 0.07	0.68 ± 0.06	0.68 ± 0.05	0.68 ± 0.04

[1] Values are percent or mean ± SD. MET, metabolic equivalent; BMI, body mass index. [2] Includes green leafy vegetables, cruciferous vegetables, dark yellow vegetables, other vegetables, and tomato food group. [3] Energy-adjusted.

Table S4. Characteristics of MESA participants according to quintiles of plant protein intake (n=5,879)[1]

	Quintiles of plant protein intake (g) measured at Exam 1				
Characteristic	Q1	Q2	Q3	Q4	Q5
n	1173	1176	1176	1178	1176
Mean (min, max)	11.3 (0.6, 14.2)	16.3 (14.2, 18.5)	20.9 (18.5, 23.4)	26.6 (23.4, 30.6)	41.2 (30.6, 118,6)
**Demographic factors**					
Age, y	63.1 ± 10.1	63.0 ± 10.6	62.6 ± 10.0	61.7 ± 10.2	61.1 ± 10.4
Male, *%*	33.5	41.3	46.7	54.1	62.4
Race/ethnicity, *%*					
White	36.3	39.7	44.7	42.8	37.7
Black/African American	30.4	25.1	24.3	26.1	23.9
Hispanic	20.5	21.3	19.0	20.1	29.5
Chinese	12.8	14.0	12.1	11.0	8.9
**Socioeconomic factors**					
Income, *%*					
< $20,000	26.3	24.6	20.1	20.9	24.1
$20,000-$49,999	40.2	37.1	35.4	37.5	36.2
≥ $50,000	33.4	38.4	44.5	41.6	39.7
Highest education level, *%*					
< High school	20.1	18.4	15.5	15.3	20.5
High school	20.6	19.8	17.8	15.7	15.0
> High school	59.3	61.8	66.7	69.0	64.5
**Lifestyle and comorbidity factors**					
Current smokers, *%*	15.0	10.9	12.2	12.7	11.6
Cigarette pack-years	12.0 ± 20.8	10.4 ± 19.6	11.7 ± 21.2	11.4 ± 19.8	11.2 ± 22.2
Moderate and vigorous physical activity, *MET-min/wk*	5054.9 ± 5895.0	5414.8 ± 5639.1	5571.2 ± 5321.5	6191.6 ± 6035.1	6428.6 ± 6525.8
Dietary supplement use, *%*	90.4	89.7	90.8	87.2	88.8
Prevalent diabetes, *%*	12.8	13.0	11.5	11.4	13.0
BMI, *kg/m²*	28.2 ± 5.6	28.2 ± 5.4	28.2 ± 5.4	28.1 ± 5.2	28.7 ± 5.3
**Dietary factors**					
Energy intake, *kcal/d*	1015.0 ± 323.5	1281.6 ± 376.6	1588.6 ± 417.3	1925.6 ± 491.1	2664.8 ± 848.4
Alcohol intake, *g/d*	4.7 ± 12.4	4.7 ± 12.7	5.3 ± 12.2	5.8 ± 11.4	6.6 ± 16.2
Fruit intake, *servings/day*	1.3 ± 1.0	1.7 ± 1.2	2.0 ± 1.4	2.3 ± 1.7	2.9 ± 2.1
Vegetable[2] intake, *servings/day*	1.4 ± 0.8	1.9 ± 1.0	2.4 ± 1.1	2.8 ± 1.3	3.9 ± 2.0
Total fiber intake, *g/d*	10.2 ± 2.9	14.4 ± 3.2	17.8 ± 3.7	22.3 ± 4.6	33.5 ± 9.8
Saturated fat intake, *% of energy*	10.6 ± 3.5	10.0 ± 3.2	10.0 ± 3.1	10.1 ± 2.9	9.5 ± 2.7
Polyunsaturated fat intake, *% of energy*	5.8 ± 1.9	5.9 ± 1.6	5.9 ± 1.6	6.2 ± 1.7	6.3 ± 1.8
Monounsaturated fat intake, *% of energy*	12.0 ± 3.1	11.7 ± 2.8	11.8 ± 2.8	12.0 ± 2.7	11.7 ± 2.6
trans fat intake, *% of energy*	0.8 ± 0.3	0.8 ± 0.3	0.8 ± 0.3	0.8 ± 0.3	0.8 ± 0.3
Vit E intake, *IU/d*	5.1 ± 2.7	6.7 ± 3.3	8.2 ± 3.4	10.0 ± 3.4	14.3 ± 6.5
Protein intake[3], *g/d*					
Total protein intake	65.1 ± 10.8	66.0 ± 11.1	65.4 ± 12.4	66.3 ± 14.5	67.8 ± 19.3
Animal protein intake	45.2 ± 11.1	44.3 ± 11.9	42.8 ± 12.8	41.9 ± 15.0	37.7 ± 19.8
Protein intake, *% of energy*					
Total protein	15.9 ± 3.9	15.9 ± 3.2	15.6 ± 3.0	15.7 ± 2.9	15.7 ± 2.8
Animal protein	11.0 ± 4.0	10.3 ± 3.3	9.9 ± 3.1	9.7 ± 3.0	9.1 ± 2.9
Plant protein	4.8 ± 1.3	5.5 ± 1.4	5.6 ± 1.4	5.9 ± 1.5	6.5 ± 1.8

Diversity of total protein foods					
Count	6.5 ± 3.0	7.6 ± 3.2	8.7 ± 3.5	9.9 ± 3.7	11.9 ± 4.6
Dissimilarity	0.67 ± 0.08	0.68 ± 0.07	0.68 ± 0.06	0.69 ± 0.05	0.69 ± 0.05

[1]Values are percent or mean ± SD. MET, metabolic equivalent; BMI, body mass index. [2] Includes green leafy vegetables, cruciferous vegetables, dark yellow vegetables, other vegetables, and tomato food group. [3] Energy-adjusted.

Table S5. Spearman partial correlations among protein exposures in 5,879 participants in MESA

	Percent energy from total protein	Percent energy from animal protein	Percent energy from plant protein
Total protein consumption (g/day)	0.91	0.81	0.11
Protein intake from animal sources (g/day)	0.80	0.93	-0.33
Protein intake from plant sources (g/day)	0.11	-0.32	0.91

*Correlations were adjusted for age, sex, race/ethnicity, and energy intake. All p-values were <0.0001.

Table S6. Hazard Ratios (95% CIs) of Incident CVD, CHD, and stroke by Quintile and Continuous Percentage of Energy Intake from Total Protein in MESA

	HR (95% CI)						
	Q1	Q2	Q3	Q4	Q5	P_{trend}	For 5% E increment
**Percentage of energy from total protein**							
**CVD (n=5,879)**							
Mean (min, max)	11.6 (4.8, 13.1)	14.0 (13.1, 14.9)	15.6 (14.9, 16.3)	17.2 (16.3, 18.2)	20.3 (18.2, 33.2)		
Cases	196	210	203	223	213		
Model 1	Reference	1.06 (0.87,1.29)	1.11 (0.92,1.36)	1.28 (1.06,1.56)	1.29 (1.05,1.57)	0.003	1.14 (1.03,1.26)
Model 2	Reference	1.06 (0.87,1.29)	1.07 (0.87,1.30)	1.21 (1.00,1.48)	1.18 (0.96,1.45)	0.05	1.07 (0.97,1.19)
Model 2-BMI, diabetes	Reference	1.08 (0.89,1.32)	1.13 (0.93,1.38)	1.30 (1.06,1.58)	1.31 (1.07,1.60)	0.002	1.14 (1.03,1.27)
Model 3a	Reference	1.07 (0.87,1.30)	1.09 (0.89,1.34)	1.24 (1.01,1.52)	1.21 (0.98,1.51)	0.04	1.09 (0.97,1.21)
Model 3b	Reference	1.05 (0.86,1.28)	1.08 (0.88,1.32)	1.21 (0.99,1.47)	1.20 (0.98,1.47)	0.03	1.09 (0.97,1.21)
**CHD (n=5,878)**							
Mean (min, max)	11.6 (4.8, 13.1)	14.0 (13.1, 14.9)	15.6 (14.9, 16.3)	17.2 (16.3, 18.2)	20.3 (18.2, 33.2)		
Cases	121	144	124	145	134		
Model 1	Reference	1.18 (0.92,1.50)	1.09 (0.85,1.40)	1.32 (1.04,1.69)	1.29 (1.00,1.66)	0.03	1.12 (0.99,1.27)
Model 2	Reference	1.18 (0.93,1.51)	1.05 (0.81,1.36)	1.25 (0.97,1.60)	1.18 (0.91,1.54)	0.20	1.05 (0.92,1.20)
Model 2-BMI, diabetes	Reference	1.21 (0.94,1.54)	1.11 (0.86,1.44)	1.35 (1.05,1.72)	1.32 (1.02,1.71)	0.02	1.13 (0.99,1.28)
Model 3a	Reference	1.20 (0.94,1.54)	1.07 (0.82,1.39)	1.28 (0.98,1.65)	1.21 (0.92,1.59)	0.18	1.06 (0.92,1.22)
Model 3b	Reference	1.17 (0.92,1.50)	1.06 (0.82,1.38)	1.24 (0.96,1.59)	1.20 (0.93,1.55)	0.17	1.07 (0.94,1.21)
**Stroke (n=5,876)**							
Mean (min, max)	11.6 (4.8, 13.1)	14.0 (13.1, 14.9)	15.6 (14.9, 16.3)	17.2 (16.3, 18.2)	20.3 (18.2, 33.2)		
Cases	66	59	63	77	67		

Model 1	Reference	0.88 (0.62,1.25)	1.00 (0.70,1.41)	1.29 (0.93,1.80)	1.21 (0.85,1.72)	0.07	1.15 (0.96,1.37)
Model 2	Reference	0.85 (0.60,1.22)	0.93 (0.66,1.33)	1.21 (0.86,1.69)	1.10 (0.77,1.57)	0.21	1.09 (0.91,1.31)
Model 2-BMI, diabetes	Reference	0.88 (0.62,1.41)	0.99 (0.70,1.41)	1.28 (0.91,1.79)	1.20 (0.85,1.71)	0.08	1.15 (0.96,1.37)
Model 3a	Reference	0.85 (0.60,1.22)	0.94 (0.65,1.34)	1.21 (0.85,1.72)	1.11 (0.76,1.62)	0.22	1.09 (0.90,1.32)
Model 3b	Reference	0.84 (0.59,1.20)	0.94 (0.66,1.34)	1.19 (0.85,1.68)	1.12 (0.79,1.60)	0.17	1.14 (0.93,1.34)

Model 1 is adjusted for age (years), sex, race/ethnicity (white, Chinese, Black, Hispanic), field center, and total energy intake(kcal) in Model 1.

Model 2 is adjusted all variables in Model 1 plus education (<high school, high school, > high school), income (<$20000, $20000-$49999, >$49999), smoking status (current, former, never) and pack-years of cigarette smoking, dietary supplement use 1 per week or more (yes/no), alcohol use (g/day), physical activity (moderate and vigorous physical activity total, metabolic equivalents per min/wk), prevalent diabetes (yes/no), and body max index (kg/m2).

Model 2 – BMI, diabetes is adjusted all variables in Model 2 excluding BMI and prevalent diabetes.

Model 3a is adjusted all variables in Model 2 plus for intakes of total dietary fiber (g/day), trans fats (% of energy), saturated fatty acids (% of energy), poly-unsaturated fatty acids (% of energy), mono-unsaturated fatty acids (% of energy), vitamin E (IU/day).

Model 3b is adjusted all variables in Model 2 plus for systolic blood pressure (mmHg), diastolic blood pressure (mmHg), triglycerides (mg/dl), HDL cholesterol (mg/dl), LDL cholesterol (mg/dl), C-reactive protein (mg/l), and interleukin-6 (pg/ml).

Table S7. Hazard Ratios (95% CIs) of Incident CVD, CHD, and stroke by Quintile and Continuous Animal Protein Intake in MESA

	HR (95% CI)						
	Q1	Q2	Q3	Q4	Q5	P_{trend}	For 20g/day increment
**Animal protein intake (grams/day)**							
**CVD (n=5,879)**							
Mean (min, max)	16.6 (0.2, 22.6)	27.4 (22.6, 32.2)	37.4 (32.2, 43.1)	49.9 (43.1, 58.5)	80.4 (58.5, 210.0)		
Cases	198	218	209	224	196		
Model 1	Reference	1.06 (0.88,1.29)	1.11 (0.91,1.37)	1.35 (1.08,1.68)	1.28 (0.97,1.68)	0.04	1.10 (1.01,1.19)
Model 2	Reference	1.06 (0.87,1.28)	1.04 (0.84,1.28)	1.25 (1.00,1.56)	1.12 (0.85,1.48)	0.28	1.04 (0.96,1.13)
Model 2-BMI, diabetes	Reference	1.06 (0.88,1.30)	1.13 (0.92,1.38)	1.34 (1.07,1.67)	1.27 (0.96,1.68)	0.05	1.09 (1.00,1.18)
Model 3a	Reference	1.07 (0.88,1.30)	1.05 (0.85,1.30)	1.27 (1.00,1.61)	1.14 (0.83,1.55)	0.30	1.04 (0.94,1.14)
Model 3b	Reference	1.10 (0.90,1.33)	1.07 (0.87,1.32)	1.29 (1.03,1.60)	1.20 (0.91,1.59)	0.14	1.05 (0.97,1.14)
**CHD (n=5,878)**							
Mean (min, max)	16.6 (0.2, 22.6)	27.4 (22.6, 32.2)	37.4 (32.2, 43.1)	49.9 (43.1, 58.5)	80.4 (58.5, 210.0)		
Cases	128	133	139	142	126		
Model 1	Reference	0.97 (0.76,1.24)	1.06 (0.82,1.36)	1.17 (0.89,1.54)	1.09 (0.78,1.54)	0.43	1.04 (0.94,1.16)
Model 2	Reference	0.96 (0.75,1.22)	0.98 (0.76,1.26)	1.08 (0.82,1.42)	0.96 (0.68,1.35)	0.99	0.99 (0.89,1.10)
Model 2-BMI, diabetes	Reference	0.96 (0.75,1.23)	1.06 (0.82,1.37)	1.16 (0.88,1.53)	1.08 (0.77,1.53)	0.46	1.04 (0.93,1.15)
Model 3a	Reference	0.96 (0.75,1.24)	0.98 (0.75,1.29)	1.09 (0.81,1.47)	0.97 (0.66,1.43)	0.96	0.98 (0.87,1.13)
Model 3b	Reference	0.99 (0.77,1.26)	1.00 (0.78,1.30)	1.09 (0.83,1.44)	1.01 (0.71,1.43)	0.83	1.00 (0.90,1.11)
**Stroke (n=5,876)**							
Mean (min, max)	16.6 (0.2, 22.6)	27.4 (22.6, 32.2)	37.4 (32.2, 43.1)	49.9 (43.1, 58.5)	80.4 (58.5, 210.0)		
Cases	65	72	66	76	53		

Model 1	Reference	1.16 (0.83,1.64)	1.21 (0.84,1.75)	1.69 (1.14,2.49)	1.45 (0.87,2.40)	0.08	1.19 (1.02,1.37)
Model 2	Reference	1.17 (0.83,1.64)	1.15 (0.80,1.67)	1.62 (1.09,2.39)	1.30 (0.78,2.17)	0.20	1.14 (0.98,1.32)
Model 2-BMI, diabetes	Reference	1.19 (0.84,1.67)	1.23 (0.85,1.77)	1.70 (1.15,2.52)	1.44 (0.86,2.39)	0.09	1.18 (1.01,1.37)
Model 3a	Reference	1.17 (0.83,1.66)	1.15 (0.78,1.68)	1.58 (1.04,2.40)	1.22 (0.69,2.15)	0.37	1.11 (0.93,1.33)
Model 3b	Reference	1.22 (0.86,1.71)	1.18 (0.82,1.71)	1.66 (1.12,2.46)	1.39 (0.83,2.32)	0.13	1.15 (0.99,1.34)

Model 1 is adjusted for age (years), sex, race/ethnicity (white, Chinese, Black, Hispanic), field center, and total energy intake(kcal) in Model 1.

Model 2 is adjusted all variables in Model 1 plus education (<high school, high school, > high school), income (<$20000, $20000-$49999, >$49999), smoking status (current, former, never) and pack-years of cigarette smoking, dietary supplement use 1 per week or more (yes/no), alcohol use (g/day), physical activity (moderate and vigorous physical activity total, metabolic equivalents per min/wk), prevalent diabetes (yes/no), and body max index (kg/m2).

Model 2 – BMI, diabetes is adjusted all variables in Model 2 excluding BMI and prevalent diabetes.

Model 3a is adjusted all variables in Model 2 plus for intakes of total dietary fiber (g/day), trans fats (% of energy), saturated fatty acids (% of energy), poly-unsaturated fatty acids (% of energy), mono-unsaturated fatty acids (% of energy), vitamin E (IU/day).

Model 3b is adjusted all variables in Model 2 plus for systolic blood pressure (mmHg), diastolic blood pressure (mmHg), triglycerides (mg/dl), HDL cholesterol (mg/dl), LDL cholesterol (mg/dl), C-reactive protein (mg/l), and interleukin-6 (pg/ml).

Table S8. Hazard Ratios (95% CIs) of Incident CVD, CHD, and stroke by Quintile and Continuous Percentage of Energy Intake from Animal Protein in MESA

	HR (95% CI)						
	Q1	Q2	Q3	Q4	Q5	P_{trend}	For 5% E increment
**Percentage of energy from animal protein**							
**CVD (n=5,879)**							
Mean (min, max)	5.7 (0.0, 7.2)	8.1 (7.2, 9.0)	9.8 (9.0, 10.6)	11.5 (10.6, 12.6)	14.9 (12.6, 30.7)		
Cases	202	200	214	220	209		
Model 1	Reference	1.04 (0.85,1.26)	1.18 (0.97,1.43)	1.29 (1.07,1.57)	1.26 (1.04,1.53)	0.003	1.13 (1.03,1.24)
Model 2	Reference	1.02 (0.84,1.24)	1.09 (0.90,1.33)	1.22 (1.00,1.47)	1.14 (0.93,1.39)	0.07	1.07 (0.97,1.17)
Model 2-BMI, diabetes	Reference	1.05 (0.86,1.27)	1.17 (0.96,1.41)	1.28 (1.06,1.56)	1.25 (1.03,1.52)	0.0005	1.13 (1.03,1.23)
Model 3a	Reference	1.03 (0.85,1.26)	1.12 (0.91,1.37)	1.24 (1.01,1.53)	1.18 (0.94,1.47)	0.06	1.08 (0.97,1.21)
Model 3b	Reference	1.01 (0.83,1.24)	1.12 (0.92,1.36)	1.22 (1.00,1.48)	1.15 (0.95,1.41)	0.05	1.08 (0.98,1.18)
**CHD (n=5,878)**							
Mean (min, max)	5.7 (0.0, 7.2)	8.1 (7.2, 9.0)	9.8 (9.0, 10.6)	11.5 (10.6, 12.6)	14.9 (12.6, 30.7)		
Cases	132	140	129	137	130		
Model 1	Reference	1.09 (0.86,1.38)	1.06 (0.83,1.36)	1.21 (0.95,1.54)	1.18 (0.92,1.50)	0.13	1.08 (0.96,1.21)
Model 2	Reference	1.07 (0.85,1.36)	0.99 (0.77,1.26)	1.13 (0.89,1.45)	1.05 (0.82,1.35)	0.61	1.01 (0.90,1.14)
Model 2-BMI, diabetes	Reference	1.10 (0.86,1.39)	1.05 (0.82,1.34)	1.20 (0.94,1.53)	1.17 (0.91,1.49)	0.16	1.07 (0.95,1.21)
Model 3a	Reference	1.09 (0.85,1.39)	1.01 (0.78,1.30)	1.16 (0.89,1.50)	1.09 (0.82,1.44)	0.48	1.02 (0.89,1.17)
Model 3b	Reference	1.06 (0.83,1.35)	0.99 (0.78,1.27)	1.13 (0.89,1.44)	1.06 (0.83,1.36)	0.55	1.02 (0.91,1.15)
**Stroke (n=5,876)**							
Mean (min, max)	5.7 (0.0, 7.2)	8.1 (7.2, 9.0)	9.8 (9.0, 10.6)	11.5 (10.6, 12.6)	14.9 (12.6, 30.7)		
Cases	61	57	74	69	71		

Model 1	Reference	0.98 (0.69,1.41)	1.34 (0.95,1.88)	1.32 (0.93,1.86)	1.43 (1.02,2.03)	0.01	1.22 (1.04,1.43)
Model 2	Reference	0.97 (0.67,1.39)	1.28 (0.91,1.80)	1.24 (0.88,1.76)	1.33 (0.94,1.89)	0.05	1.17 (0.99,1.38)
Model 2-BMI, diabetes	Reference	0.99 (0.69,1.42)	1.34 (0.95,1.88)	1.31 (0.92,1.85)	1.43 (1.01,2.02)	0.02	1.22 (1.04,1.43)
Model 3a	Reference	0.96 (0.66,1.39)	1.27 (0.89,1.82)	1.23 (0.85,1.78)	1.31 (0.88,1.94)	0.10	1.16 (0.96,1.40)
Model 3b	Reference	0.95 (0.66,1.37)	1.30 (0.92,1.83)	1.23 (0.87,1.74)	1.34 (0.95,1.91)	0.04	1.18 (1.01,1.39)

Model 1 is adjusted for age (years), sex, race/ethnicity (white, Chinese, Black, Hispanic), field center, and total energy intake(kcal) in Model 1.

Model 2 is adjusted all variables in Model 1 plus education (<high school, high school, > high school), income (<$20000, $20000-$49999, >$49999), smoking status (current, former, never) and pack-years of cigarette smoking, dietary supplement use 1 per week or more (yes/no), alcohol use (g/day), physical activity (moderate and vigorous physical activity total, metabolic equivalents per min/wk), prevalent diabetes (yes/no), and body max index (kg/m2).

Model 2 – BMI, diabetes is adjusted all variables in Model 2 excluding BMI and prevalent diabetes.

Model 3a is adjusted all variables in Model 2 plus for intakes of total dietary fiber (g/day), trans fats (% of energy), saturated fatty acids (% of energy), poly-unsaturated fatty acids (% of energy), mono-unsaturated fatty acids (% of energy), vitamin E (IU/day).

Model 3b is adjusted all variables in Model 2 plus for systolic blood pressure (mmHg), diastolic blood pressure (mmHg), triglycerides (mg/dl), HDL cholesterol (mg/dl), LDL cholesterol (mg/dl), C-reactive protein (mg/l), and interleukin-6 (pg/ml).

Table S9. Hazard Ratios (95% CIs) of Incident CVD, CHD, and stroke by Quintile and Continuous Plant Protein Intake in MESA

	HR (95% CI)						
	Q1	Q2	Q3	Q4	Q5	P_{trend}	For 20g/day increment
**Plant protein intake (grams/day)**							
**CVD (n=5,879)**							
Mean (min, max)	11.3 (0.6, 14.2)	16.3 (14.2, 18.5)	20.9 (18.5, 23.4)	26.6 (23.4, 30.6)	41.2 (30.6, 118.6)		
Cases	209	216	206	206	208		
Model 1	Reference	0.97 (0.80,1.18)	0.89 (0.72,1.09)	0.89 (0.71,1.11)	0.88 (0.66,1.16)	0.34	0.95 (0.79,1.15)
Model 2	Reference	1.01 (0.83,1.23)	0.94 (0.77,1.16)	0.95 (0.76,1.20)	0.95 (0.71,1.26)	0.66	0.99 (0.81,1.20)
Model 2-BMI, diabetes	Reference	1.01 (0.83,1.23)	0.93 (0.76,1.15)	0.93 (0.74,1.17)	0.92 (0.69,1.23)	0.50	0.98 (0.81,1.19)
Model 3a	Reference	1.02 (0.83,1.24)	0.95 (0.76,1.19)	0.97 (0.75,1.26)	0.99 (0.68,1.45)	0.92	1.15 (0.81,1.64)
Model 3b	Reference	1.05 (0.86,1.28)	1.08 (0.88,1.32)	1.21 (0.99,1.47)	1.20 (0.98,1.47)	0.03	1.09 (0.97,1.21)
**CHD (n=5,878)**							
Mean (min, max)	11.3 (0.6, 14.2)	16.3 (14.2, 18.5)	20.9 (18.5, 23.4)	26.6 (23.4, 30.6)	41.2 (30.6, 118.6)		
Cases	125	136	127	142	138		
Model 1	Reference	1.01 (0.79,1.29)	0.89 (0.69,1.16)	0.99 (0.75,1.31)	0.94 (0.66,1.33)	0.76	1.07 (0.85,1.35)
Model 2	Reference	1.05 (0.82,1.35)	0.95 (0.73,1.24)	1.07 (0.81,1.43)	1.03 (0.72,1.47)	0.88	1.12 (0.88,1.42)
Model 2-BMI, diabetes	Reference	1.05 (0.82,1.35)	0.94 (0.72,1.23)	1.04 (0.79,1.39)	1.00 (0.70,1.43)	0.99	1.11 (0.87,1.41)
Model 3a	Reference	1.04 (0.81,1.34)	0.93 (0.70,1.23)	1.04 (0.76,1.44)	0.98 (0.62,1.55)	0.94	1.32 (0.86,2.02)
Model 3b	Reference	1.07 (0.84,1.38)	0.97 (0.74,1.26)	1.09 (0.82,1.45)	1.03 (0.72,1.48)	0.88	1.13 (0.89,1.43)
**Stroke (n=5,876)**							
Mean (min, max)	11.3 (0.6, 14.2)	16.3 (14.2, 18.5)	20.9 (18.5, 23.4)	26.6 (23.4, 30.6)	41.2 (30.6, 118.6)		
Cases	78	76	64	58	56		

Model 1	Reference	0.94 (0.68,1.30)	0.81 (0.57,1.16)	0.73 (0.49,1.09)	0.71 (0.43,1.19)	0.15	0.72 (0.50,1.04)
Model 2	Reference	0.95 (0.68,1.31)	0.83 (0.58,1.20)	0.74 (0.49,1.11)	0.70 (0.41,1.18)	0.14	0.70 (0.48,1.01)
Model 2-BMI, diabetes	Reference	0.95 (0.68,1.31)	0.82 (0.57,1.17)	0.72 (0.48,1.09)	0.68 (0.41,1.16)	0.11	0.69 (0.48,1.00)
Model 3a	Reference	0.97 (0.69,1.35)	0.86 (0.58,1.28)	0.79 (0.49,1.27)	0.80 (0.40,1.61)	0.45	0.63 (0.32,1.24)
Model 3b	Reference	0.98 (0.71,1.36)	0.88 (0.61,1.27)	0.75 (0.50,1.13)	0.72 (0.43,1.22)	0.15	0.71 (0.49,1.03)

Model 1 is adjusted for age (years), sex, race/ethnicity (white, Chinese, Black, Hispanic), field center, and total energy intake(kcal) in Model 1.

Model 2 is adjusted all variables in Model 1 plus education (<high school, high school, > high school), income (<$20000, $20000-$49999, >$49999), smoking status (current, former, never) and pack-years of cigarette smoking, dietary supplement use 1 per week or more (yes/no), alcohol use (g/day), physical activity (moderate and vigorous physical activity total, metabolic equivalents per min/wk), prevalent diabetes (yes/no), and body max index (kg/m2).

Model 2 – BMI, diabetes is adjusted all variables in Model 2 excluding BMI and prevalent diabetes.

Model 3a is adjusted all variables in Model 2 plus for intakes of total dietary fiber (g/day), trans fats (% of energy), saturated fatty acids (% of energy), poly-unsaturated fatty acids (% of energy), mono-unsaturated fatty acids (% of energy), vitamin E (IU/day).

Model 3b is adjusted all variables in Model 2 plus for systolic blood pressure (mmHg), diastolic blood pressure (mmHg), triglycerides (mg/dl), HDL cholesterol (mg/dl), LDL cholesterol (mg/dl), C-reactive protein (mg/l), and interleukin-6 (pg/ml).

Table S10. Hazard Ratios (95% CIs) of Incident CVD, CHD, and stroke by Quintile and Continuous Percentage of Energy Intake from Plant Protein in MESA

	HR (95% CI)						
	Q1	Q2	Q3	Q4	Q5	P_{trend}	For 5% E increment
**Percentage of energy from plant protein**							
**CVD (n=5,879)**							
Mean (min, max)	3.7 (0.4, 4.3)	4.7 (4.3, 5.1)	5.5 (5.1, 5.9)	6.3 (5.9, 6.8)	8.0 (6.8, 16.2)		
Cases	211	192	228	213	201		
Model 1	Reference	0.84 (0.69,1.02)	0.96 (0.79,1.16)	0.89 (0.73,1.08)	0.84 (0.68,1.04)	0.20	0.93 (0.75,1.15)
Model 2	Reference	0.88 (0.72,1.07)	0.99 (0.81,1.20)	0.93 (0.76,1.14)	0.89 (0.72,1.10)	0.42	0.97 (0.78,1.21)
Model 2-BMI, diabetes	Reference	0.86 (0.90,1.04)	1.00 (0.82,1.21)	0.92 (0.75,1.12)	0.87 (0.70,1.07)	0.33	0.96 (0.77,1.20)
Model 3a	Reference	0.88 (0.71,1.08)	0.98 (0.79,1.23)	0.92 (0.72,1.18)	0.88 (0.64,1.19)	0.52	1.05 (0.74,1.48)
Model 3b	Reference	0.89 (0.73,1.09)	1.00 (0.82,1.21)	0.94 (0.77,1.15)	0.90 (0.73,1.11)	0.47	0.99 (0.79,1.23)
**CHD (n=5,878)**							
Mean (min, max)	3.7 (0.4, 4.3)	4.7 (4.3, 5.1)	5.5 (5.1, 5.9)	6.3 (5.9, 6.8)	8.0 (6.8, 16.2)		
Cases	136	119	138	137	138		
Model 1	Reference	0.80 (0.63,1.03)	0.91 (0.71,1.15)	0.90 (0.71,1.15)	0.92 (0.71,1.18)	0.81	1.11 (0.85,1.44)
Model 2	Reference	0.85 (0.66,1.08)	0.94 (0.73,1.20)	0.96 (0.75,1.23)	0.97 (0.75,1.26)	0.87	1.17 (0.90,1.53)
Model 2-BMI, diabetes	Reference	0.82 (0.64,1.06)	0.94 (0.74,1.20)	0.94 (0.74,1.21)	0.95 (0.73,1.23)	0.94	1.16 (0.89,1.52)
Model 3a	Reference	0.84 (0.65,1.09)	0.92 (0.70,1.22)	0.93 (0.68,1.27)	0.92 (0.62,1.34)	0.88	1.33 (0.87,2.03)
Model 3b	Reference	0.86 (0.67,1.10)	0.95 (0.74,1.21)	0.97 (0.75,1.24)	0.98 (0.76,1.28)	0.81	1.19 (0.91,1.56)
**Stroke (n=5,876)**							
Mean (min, max)	3.7 (0.4, 4.3)	4.7 (4.3, 5.1)	5.5 (5.1, 5.9)	6.3 (5.9, 6.8)	8.0 (6.8, 16.2)		
Cases	69	68	78	63	54		

Model 1	Reference	0.91 (0.65,1.28)	0.97 (0.70,1.35)	0.78 (0.55,1.11)	0.67 (0.46,0.97)	0.03	0.64 (0.43,0.95)
Model 2	Reference	0.93 (0.66,1.31)	0.96 (0.69,1.34)	0.77 (0.54,1.10)	0.66 (0.45,0.97)	0.02	0.62 (0.41,0.93)
Model 2-BMI, diabetes	Reference	0.91 (0.65,1.27)	0.97 (0.69,1.35)	0.76 (0.53,1.09)	0.65 (0.44,0.95)	0.02	0.62 (0.41,0.92)
Model 3a	Reference	0.89 (0.62,1.28)	0.90 (0.62,1.32)	0.70 (0.45,1.09)	0.59 (0.34,1.02)	0.04	0.50 (0.26,0.97)
Model 3b	Reference	0.95 (0.68,1.34)	0.98 (0.70,1.37)	0.79 (0.55,1.12)	0.68 (0.46,1.00)	0.03	0.64 (0.43,0.96)

Model 1 is adjusted for age (years), sex, race/ethnicity (white, Chinese, Black, Hispanic), field center, and total energy intake(kcal) in Model 1.

Model 2 is adjusted all variables in Model 1 plus education (<high school, high school, > high school), income (<$20000, $20000-$49999, >$49999), smoking status (current, former, never) and pack-years of cigarette smoking, dietary supplement use 1 per week or more (yes/no), alcohol use (g/day), physical activity (moderate and vigorous physical activity total, metabolic equivalents per min/wk), prevalent diabetes (yes/no), and body max index (kg/m2).

Model 2 – BMI, diabetes is adjusted all variables in Model 2 excluding BMI and prevalent diabetes.

Model 3a is adjusted all variables in Model 2 plus for intakes of total dietary fiber (g/day), trans fats (% of energy), saturated fatty acids (% of energy), poly-unsaturated fatty acids (% of energy), mono-unsaturated fatty acids (% of energy), vitamin E (IU/day).

Model 3b is adjusted all variables in Model 2 plus for systolic blood pressure (mmHg), diastolic blood pressure (mmHg), triglycerides (mg/dl), HDL cholesterol (mg/dl), LDL cholesterol (mg/dl), C-reactive protein (mg/l), and interleukin-6 (pg/ml).

Table S11. Hazard Ratios (95% CIs) of Incident CVD, CHD, and stroke by Quintile and Continuous Count of Total Protein Sources in MESA

	HR (95% CI)						
	Q1	Q2	Q3	Q4	Q5	P_{trend}	For 1 count increment
Count of total protein sources							
**CVD (n=5,879)**							
Mean (min, max)	3.8 (0.0, 5.0)	6.5 (6.0, 7.0)	8.5 (8.0, 9.0)	10.9 (10.0, 12.0)	15.5 (13.0, 31.0)		
Cases	190	211	205	249	190		
Model 1	Reference	0.99 (0.82,1.21)	1.10 (0.90,1.35)	1.15 (0.94,1.40)	1.21 (0.96,1.53)	0.05	1.02 (1.00,1.04)
Model 2	Reference	0.99 (0.81,1.20)	1.09 (0.89,1.33)	1.14 (0.93,1.39)	1.20 (0.95,1.51)	0.05	1.02 (1.00,1.04)
Model 3a	Reference	0.99 (0.81,1.21)	1.09 (0.89,1.34)	1.14 (0.93,1.40)	1.20 (0.95,1.53)	0.05	1.02 (1.00,1.04)
Model 3b	Reference	1.01 (0.83,1.23)	1.08 (0.88,1.32)	1.14 (0.93,1.39)	1.22 (0.97,1.54)	0.05	1.02 (1.00,1.04)
**CHD (n=5,878)**							
Mean (min, max)	3.8 (0.0, 5.0)	6.5 (6.0, 7.0)	8.5 (8.0, 9.0)	10.9 (10.0, 12.0)	15.5 (13.0, 31.0)		
Cases	120	131	131	165	121		
Model 1	Reference	0.97 (0.76,1.25)	1.08 (0.83,1.39)	1.17 (0.91,1.50)	1.19 (0.89,1.59)	0.09	1.02 (1.00,1.05)
Model 2	Reference	0.95 (0.74,1.22)	1.06 (0.82,1.37)	1.16 (0.91,1.49)	1.18 (0.88,1.58)	0.10	1.02 (1.00,1.04)
Model 3a	Reference	0.95 (0.74,1.23)	1.05 (0.81,1.36)	1.15 (0.89,1.49)	1.17 (0.87,1.58)	0.11	1.02 (1.00,1.04)
Model 3b	Reference	0.98 (0.76,1.26)	1.06 (0.82,1.36)	1.16 (0.90,1.49)	1.20 (0.90,1.61)	0.10	1.02 (1.00,1.05)
**Stroke (n=5,876)**							
Mean (min, max)	3.8 (0.0, 5.0)	6.5 (6.0, 7.0)	8.5 (8.0, 9.0)	10.9 (10.0, 12.0)	15.5 (13.0, 31.0)		
Cases	59	71	79	69	54		
Model 1	Reference	1.13 (0.80,1.61)	1.48 (1.04,2.09)	1.14 (0.79,1.64)	1.28 (0.84,1.96)	0.41	1.02 (0.99,1.06)
Model 2	Reference	1.13 (0.80,1.61)	1.43 (1.01,2.03)	1.10 (0.76,1.60)	1.23 (0.80,1.89)	0.55	1.02 (0.98,1.05)

Model 3a	Reference	1.15 (0.81,1.64)	1.48 (1.04,2.10)	1.15 (0.79,1.67)	1.29 (0.83,1.98)	0.44	1.02 (0.99,1.06)
Model 3b	Reference	1.16 (0.82,1.65)	1.40 (0.99,1.98)	1.11 (0.77,1.60)	1.25 (0.81,1.91)	0.54	1.02 (0.99,1.05)

Model 1 is adjusted for age (years), sex, race/ethnicity (white, Chinese, Black, Hispanic), field center, and total energy intake(kcal) in Model 1.

Model 2 is adjusted all variables in Model 1 plus education (<high school, high school, > high school), income (<$20000, $20000-$49999, >$49999), smoking status (current, former, never) and pack-years of cigarette smoking, dietary supplement use 1 per week or more (yes/no), alcohol use (g/day), physical activity (moderate and vigorous physical activity total, metabolic equivalents per min/wk), prevalent diabetes (yes/no), and body max index (kg/m2).

Model 3a is adjusted all variables in Model 2 plus for intakes of total dietary fiber (g/day), trans fats (% of energy), saturated fatty acids (% of energy), poly-unsaturated fatty acids (% of energy), mono-unsaturated fatty acids (% of energy), vitamin E (IU/day).

Model 3b is adjusted all variables in Model 2 plus for systolic blood pressure (mmHg), diastolic blood pressure (mmHg), triglycerides (mg/dl), HDL cholesterol (mg/dl), LDL cholesterol (mg/dl), C-reactive protein (mg/l), and interleukin-6 (pg/ml).

Table S12. Hazard Ratios (95% CIs) of Incident CVD, CHD, and stroke by Quintile and Continuous Count of Animal Protein Sources in MESA

	HR (95% CI)						
	Q1	Q2	Q3	Q4	Q5	P_{trend}	For 1 count increment
Count of animal protein sources							
**CVD (n=5,879)**							
Mean (min, max)	2.3 (0.0, 3.0)	4.5 (4.0, 5.0)	6.5 (6.0, 7.0)	8.4 (8.0, 9.0)	12.0 (10.0, 22.0)		
Cases	143	252	265	185	200		
Model 1	Reference	1.08 (0.88,1.33)	1.19 (0.97,1.47)	1.12 (0.89,1.40)	1.27 (1.00,1.60)	0.08	1.02 (1.00,1.04)
Model 2	Reference	1.04 (0.85,1.28)	1.15 (0.93,1.41)	1.06 (0.84,1.33)	1.20 (0.94,1.52)	0.19	1.02 (0.99,1.04)
Model 3a	Reference	1.04 (0.85,1.28)	1.14 (0.92,1.41)	1.05 (0.83,1.33)	1.18 (0.93,1.51)	0.24	1.02 (0.99,1.04)
Model 3b	Reference	1.07 (0.87,1.32)	1.16 (0.94,1.43)	1.05 (0.84,1.32)	1.21 (0.95,1.54)	0.20	1.02 (1.00,1.04)
**CHD (n=5,878)**							
Mean (min, max)	2.3 (0.0, 3.0)	4.5 (4.0, 5.0)	6.5 (6.0, 7.0)	8.4 (8.0, 9.0)	12.0 (10.0, 22.0)		
Cases	96	151	172	123	126		
Model 1	Reference	0.95 (0.74,1.23)	1.11 (0.86,1.43)	1.05 (0.80,1.39)	1.15 (0.85,1.54)	0.27	1.01 (0.99,1.04)
Model 2	Reference	0.91 (0.70,1.17)	1.05 (0.81,1.36)	0.99 (0.75,1.32)	1.08 (0.81,1.46)	0.44	1.01 (0.98,1.04)
Model 3a	Reference	0.90 (0.69,1.17)	1.05 (0.81,1.37)	0.99 (0.75,1.32)	1.08 (0.80,1.47)	0.45	1.01 (0.98,1.04)
Model 3b	Reference	0.92 (0.71,1.19)	1.06 (0.82,1.37)	0.99 (0.75,1.30)	1.09 (0.81,1.46)	0.47	1.01 (0.98,1.04)
**Stroke (n=5,876)**							
Mean (min, max)	2.3 (0.0, 3.0)	4.5 (4.0, 5.0)	6.5 (6.0, 7.0)	8.4 (8.0, 9.0)	12.0 (10.0, 22.0)		
Cases	38	96	85	55	58		
Model 1	Reference	1.60 (1.09,2.33)	1.55 (1.05,2.03)	1.36 (0.89,2.09)	1.57 (1.00,2.46)	0.25	1.03 (0.99,1.07)
Model 2	Reference	1.56 (1.07,2.27)	1.50 (1.02,2.22)	1.29 (0.84,1.98)	1.47 (0.94,2.31)	0.42	1.03 (0.99,1.07)

Model 3a	Reference	1.57 (1.07,2.29)	1.50 (1.01,2.23)	1.28 (0.83,1.99)	1.44 (0.91,2.30)	0.51	1.02 (0.98,1.07)
Model 3b	Reference	1.61 (1.10,2.35)	1.51 (1.02,2.24)	1.29 (0.84,1.98)	1.49 (0.95,2.34)	0.43	1.03 (0.99,1.07)

Model 1 is adjusted for age (years), sex, race/ethnicity (white, Chinese, Black, Hispanic), field center, and total energy intake(kcal) in Model 1.

Model 2 is adjusted all variables in Model 1 plus education (<high school, high school, > high school), income (<$20000, $20000-$49999, >$49999), smoking status (current, former, never) and pack-years of cigarette smoking, dietary supplement use 1 per week or more (yes/no), alcohol use (g/day), physical activity (moderate and vigorous physical activity total, metabolic equivalents per min/wk), prevalent diabetes (yes/no), and body max index (kg/m2).

Model 3a is adjusted all variables in Model 2 plus for intakes of total dietary fiber (g/day), trans fats (% of energy), saturated fatty acids (% of energy), poly-unsaturated fatty acids (% of energy), mono-unsaturated fatty acids (% of energy), vitamin E (IU/day).

Model 3b is adjusted all variables in Model 2 plus for systolic blood pressure (mmHg), diastolic blood pressure (mmHg), triglycerides (mg/dl), HDL cholesterol (mg/dl), LDL cholesterol (mg/dl), C-reactive protein (mg/l), and interleukin-6 (pg/ml).

Table S13. Hazard Ratios (95% CIs) of Incident CVD, CHD, and stroke by Quintile and Continuous Count of Plant Protein Sources in MESA

	HR (95% CI)						
	Q1	Q2	Q3	Q4	Q5	P_{trend}	For 1 count increment
Count of plant protein sources							
**CVD (n=5,879)**							
Mean (min, max)	0.0 (0.0, 0.0)	1.0 (1.0, 1.0)	2.0 (2.0, 2.0)	3.0 (3.0, 3.0)	4.7 (4.0, 10.0)		
Cases	133	227	274	205	206		
Model 1	Reference	0.96 (0.77,1.19)	1.02 (0.83,1.26)	1.05 (0.84,1.32)	1.09 (0.87,1.38)	0.24	1.03 (0.99,1.08)
Model 2	Reference	0.98 (0.79,1.22)	1.06 (0.86,1.31)	1.09 (0.87,1.37)	1.17 (0.93,1.49)	0.09	1.04 (1.00,1.09)
Model 3a	Reference	0.99 (0.80,1.23)	1.08 (0.87,1.34)	1.12 (0.89,1.41)	1.26 (0.98,1.62)	0.03	1.06 (1.01,1.12)
Model 3b	Reference	0.97 (0.78,1.20)	1.07 (0.86,1.32)	1.09 (0.87,1.37)	1.19 (0.94,1.51)	0.06	1.05 (1.00,1.09)
**CHD (n=5,878)**							
Mean (min, max)	0.0 (0.0, 0.0)	1.0 (1.0, 1.0)	2.0 (2.0, 2.0)	3.0 (3.0, 3.0)	4.7 (4.0, 10.0)		
Cases	80	141	171	126	150		
Model 1	Reference	1.00 (0.76,1.32)	1.07 (0.82,1.40)	1.10 (0.82,1.47)	1.34 (1.00,1.79)	0.02	1.07 (1.02,1.13)
Model 2	Reference	1.03 (0.78,1.35)	1.13 (0.86,1.48)	1.15 (0.86,1.54)	1.46 (1.09,1.96)	0.005	1.09 (1.03,1.15)
Model 3a	Reference	1.04 (0.79,1.37)	1.14 (0.86,1.50)	1.17 (0.87,1.58)	1.54 (1.13,2.11)	0.004	1.11 (1.04,1.17)
Model 3b	Reference	1.02 (0.77,1.34)	1.14 (0.87,1.49)	1.16 (0.87,1.55)	1.48 (1.10,1.99)	0.003	1.09 (1.04,1.15)
**Stroke (n=5,876)**							
Mean (min, max)	0.0 (0.0, 0.0)	1.0 (1.0, 1.0)	2.0 (2.0, 2.0)	3.0 (3.0, 3.0)	4.7 (4.0, 10.0)		
Cases	40	80	93	65	54		
Model 1	Reference	1.12 (0.77,1.64)	1.16 (0.80,1.69)	1.16 (0.77,1.74)	1.02 (0.66,1.57)	0.96	0.99 (0.92,1.07)
Model 2	Reference	1.12 (0.76,1.64)	1.15 (0.79,1.68)	1.16 (0.77,1.75)	1.02 (0.66,1.58)	0.95	1.00 (0.92,1.08)

Model 3a	Reference	1.14 (0.78,1.67)	1.20 (0.82,1.76)	1.26 (0.83,1.91)	1.18 (0.74,1.88)	0.43	1.03 (0.94,1.12)
Model 3b	Reference	1.10 (0.75,1.62)	1.15 (0.79,1.68)	1.17 (0.77,1.76)	1.03 (0.67,1.60)	0.85	1.00 (0.92,1.08)

Model 1 is adjusted for age (years), sex, race/ethnicity (white, Chinese, Black, Hispanic), field center, and total energy intake(kcal) in Model 1.

Model 2 is adjusted all variables in Model 1 plus education (<high school, high school, > high school), income (<$20000, $20000-$49999, >$49999), smoking status (current, former, never) and pack-years of cigarette smoking, dietary supplement use 1 per week or more (yes/no), alcohol use (g/day), physical activity (moderate and vigorous physical activity total, metabolic equivalents per min/wk), prevalent diabetes (yes/no), and body max index (kg/m2).

Model 3a is adjusted all variables in Model 2 plus for intakes of total dietary fiber (g/day), trans fats (% of energy), saturated fatty acids (% of energy), poly-unsaturated fatty acids (% of energy), mono-unsaturated fatty acids (% of energy), vitamin E (IU/day).

Model 3b is adjusted all variables in Model 2 plus for systolic blood pressure (mmHg), diastolic blood pressure (mmHg), triglycerides (mg/dl), HDL cholesterol (mg/dl), LDL cholesterol (mg/dl), C-reactive protein (mg/l), and interleukin-6 (pg/ml).

Table S14. Hazard Ratios (95% CIs) of Incident CVD, CHD, and stroke by Quintile and Continuous Dissimilarity of Total Protein Sources in MESA

	HR (95% CI)						
	Q1	Q2	Q3	Q4	Q5	P_{trend}	For 0.01 unit increment
Dissimilarity of total protein sources							
**CVD (n=5,823)**							
Mean (min, max)	0.59 (0.00, 0.64)	0.66 (0.64, 0.67)	0.69 (0.67, 0.70)	0.71 (0.70, 0.72)	0.76 (0.72, 1.00)		
Cases	176	213	216	215	216		
Model 1	Reference	1.15 (0.94,1.41)	1.19 (0.97,1.46)	1.11 (0.91,1.36)	1.09 (0.89,1.33)	0.51	0.83 (0.32,2.18)
Model 2	Reference	1.15 (0.94,1.41)	1.16 (0.95,1.43)	1.13 (0.92,1.39)	1.11 (0.91,1.36)	0.35	0.91 (0.34,2.45)
Model 3a	Reference	1.15 (0.94,1.41)	1.18 (0.96,1.44)	1.15 (0.94,1.41)	1.13 (0.92,1.39)	0.27	0.95 (0.35,2.60)
Model 3b	Reference	1.14 (0.93,1.39)	1.15 (0.94,1.41)	1.14 (0.93,1.39)	1.11 (0.90,1.36)	0.36	0.89 (0.33,2.40)
**CHD (n=5,820)**							
Mean (min, max)	0.59 (0.00, 0.64)	0.66 (0.64, 0.67)	0.69 (0.67, 0.70)	0.71 (0.70, 0.72)	0.76 (0.72, 1.00)		
Cases	110	131	136	153	134		
Model 1	Reference	1.14 (0.88,1.47)	1.20 (0.93,1.56)	1.29 (1.00,1.66)	1.11 (0.86,1.43)	0.26	1.85 (0.53,6.48)
Model 2	Reference	1.13 (0.88,1.46)	1.19 (0.92,1.53)	1.33 (1.03,1.71)	1.14 (0.88,1.47)	0.17	2.13 (0.59,7.72)
Model 3a	Reference	1.12 (0.87,1.45)	1.19 (0.92,1.54)	1.33 (1.03,1.72)	1.14 (0.88,1.48)	0.17	2.10 (0.57,7.78)
Model 3b	Reference	1.12 (0.86,1.44)	1.16 (0.90,1.50)	1.32 (1.03,1.70)	1.13 (0.87,1.46)	0.18	2.03 (0.56,7.35)
**Stroke (n=5,820)**							
Mean (min, max)	0.59 (0.00, 0.64)	0.66 (0.64, 0.67)	0.69 (0.67, 0.70)	0.71 (0.70, 0.72)	0.76 (0.72, 1.00)		
Cases	59	65	66	66	71		
Model 1	Reference	1.05 (0.74,1.50)	1.06 (0.74,1.52)	1.00 (0.70,1.44)	1.00 (0.70,1.42)	0.93	0.45 (0.09,2.29)

Model 2	Reference	1.06 (0.74,1.52)	1.03 (0.72,1.48)	1.00 (0.69,1.43)	1.01 (0.71,1.43)	0.93	0.44 (0.08,2.27)
Model 3a	Reference	1.08 (0.76,1.55)	1.06 (0.74,1.52)	1.03 (0.72,1.49)	1.05 (0.73,1.50)	0.88	0.52 (0.10,2.80)
Model 3b	Reference	1.04 (0.73,1.49)	1.01 (0.71,1.45)	0.99 (0.69,1.42)	1.00 (0.71,1.43)	0.94	0.44 (0.08,2.29)

*Hazard ratios for 20g/day increase in protein intake measures or 5% increase in energy from protein intake measures.

Model 1 is adjusted for age (years), sex, race/ethnicity (white, Chinese, Black, Hispanic), field center, and total energy intake(kcal) in Model 1.

Model 2 is adjusted all variables in Model 1 plus education (<high school, high school, > high school), income (<$20000, $20000-$49999, >$49999), smoking status (current, former, never) and pack-years of cigarette smoking, dietary supplement use 1 per week or more (yes/no), alcohol use (g/day), physical activity (moderate and vigorous physical activity total, metabolic equivalents per min/wk), prevalent diabetes (yes/no), and body max index (kg/m2).

Model 3a is adjusted all variables in Model 2 plus for intakes of total dietary fiber (g/day), trans fats (% of energy), saturated fatty acids (% of energy), poly-unsaturated fatty acids (% of energy), mono-unsaturated fatty acids (% of energy), vitamin E (IU/day).

Model 3b is adjusted all variables in Model 2 plus for systolic blood pressure (mmHg), diastolic blood pressure (mmHg), triglycerides (mg/dl), HDL cholesterol (mg/dl), LDL cholesterol (mg/dl), C-reactive protein (mg/l), and interleukin-6 (pg/ml).

Table S15. Hazard Ratios (95% CIs) of Incident CVD, CHD, and stroke by Quintile and Continuous Dissimilarity of Animal Protein Sources in MESA

	HR (95% CI)						
	Q1	Q2	Q3	Q4	Q5	P_{trend}	For 0.01 unit increment
Dissimilarity of animal protein sources							
**CVD (n=5,732)**							
Mean (min, max)	0.51 (0.00, 0.58)	0.60 (0.58, 0.62)	0.63 (0.62, 0.65)	0.66 (0.65, 0.68)	0.71 (0.68, 0.92)		
Cases	209	171	226	205	212		
Model 1	Reference	0.80 (0.65,0.98)	1.08 (0.89,1.31)	0.95 (0.78,1.16)	0.93 (0.77,1.13)	0.87	1.02 (0.46,2.27)
Model 2	Reference	0.76 (0.62,0.94)	1.07 (0.88,1.30)	0.92 (0.75,1.12)	0.93 (0.76,1.13)	0.86	0.92 (0.41,2.08)
Model 3a	Reference	0.75 (0.61,0.92)	1.07 (0.88,1.29)	0.91 (0.75,1.11)	0.92 (0.75,1.12)	0.81	0.89 (0.39,2.03)
Model 3b	Reference	0.76 (0.62,0.93)	1.08 (0.89,1.31)	0.92 (0.76,1.13)	0.93 (0.77,1.14)	0.94	0.96 (0.42,2.18)
**CHD (n=5,729)**							
Mean (min, max)	0.51 (0.00, 0.58)	0.60 (0.58, 0.62)	0.63 (0.62, 0.65)	0.66 (0.65, 0.68)	0.71 (0.68, 0.92)		
Cases	131	103	147	141	130		
Model 1	Reference	0.76 (0.59,0.99)	1.11 (0.87,1.42)	1.04 (0.82,1.33)	0.92 (0.72,1.18)	0.84	1.31 (0.47,3.69)
Model 2	Reference	0.73 (0.56,0.95)	1.11 (0.87,1.41)	1.02 (0.80,1.30)	0.92 (0.72,1.18)	0.83	1.21 (0.42,3.48)
Model 3a	Reference	0.72 (0.55,0.93)	1.11 (0.87,1.42)	1.01 (0.79,1.29)	0.91 (0.71,1.17)	0.87	1.16 (0.40,3.37)
Model 3b	Reference	0.73 (0.56,0.94)	1.11 (0.87,1.42)	1.02 (0.80,1.30)	0.93 (0.72,1.19)	0.77	1.25 (0.43,3.58)
**Stroke (n=5,729)**							
Mean (min, max)	0.51 (0.00, 0.58)	0.60 (0.58, 0.62)	0.63 (0.62, 0.65)	0.66 (0.65, 0.68)	0.71 (0.68, 0.92)		
Cases	69	56	71	59	71		
Model 1	Reference	0.82 (0.57,1.17)	1.03 (0.73,1.45)	0.83 (0.58,1.18)	0.92 (0.66,1.29)	0.63	0.65 (0.17,2.54)

Model 2	Reference	0.79 (0.55,1.13)	1.00 (0.71,1.40)	0.80 (0.56,1.14)	0.91 (0.65,1.27)	0.58	0.59 (0.15,2.32)
Model 3a	Reference	0.79 (0.55,1.13)	0.99 (0.70,1.39)	0.80 (0.56,1.14)	0.91 (0.65,1.28)	0.60	0.61 (0.15,2.44)
Model 3b	Reference	0.77 (0.54,1.10)	0.99 (0.70,1.39)	0.80 (0.56,1.14)	0.91 (0.65,1.28)	0.64	0.64 (0.16,2.52)

*Hazard ratios for 20g/day increase in protein intake measures or 5% increase in energy from protein intake measures.

Model 1 is adjusted for age (years), sex, race/ethnicity (white, Chinese, Black, Hispanic), field center, and total energy intake(kcal) in Model 1.

Model 2 is adjusted all variables in Model 1 plus education (<high school, high school, > high school), income (<$20000, $20000-$49999, >$49999), smoking status (current, former, never) and pack-years of cigarette smoking, dietary supplement use 1 per week or more (yes/no), alcohol use (g/day), physical activity (moderate and vigorous physical activity total, metabolic equivalents per min/wk), prevalent diabetes (yes/no), and body max index (kg/m2).

Model 3a is adjusted all variables in Model 2 plus for intakes of total dietary fiber (g/day), trans fats (% of energy), saturated fatty acids (% of energy), poly-unsaturated fatty acids (% of energy), mono-unsaturated fatty acids (% of energy), vitamin E (IU/day).

Model 3b is adjusted all variables in Model 2 plus for systolic blood pressure (mmHg), diastolic blood pressure (mmHg), triglycerides (mg/dl), HDL cholesterol (mg/dl), LDL cholesterol (mg/dl), C-reactive protein (mg/l), and interleukin-6 (pg/ml).

Table S16. Hazard Ratios (95% CIs) of Incident CVD, CHD, and stroke by Quintile and Continuous Dissimilarity of Plant Protein Sources in MESA

	HR (95% CI)						
	Q1	Q2	Q3	Q4	Q5	P_{trend}	For 0.01 unit increment
Dissimilarity of plant protein sources							
**CVD (n=3,693)**							
Mean (min, max)	0.24 (0.00, 0.40)	0.44 (0.41, 0.46)	0.54 (0.46, 0.57)	0.61 (0.57, 0.63)	0.75 (0.63, 1.00)		
Cases	121	146	192	121	105		
Model 1	Reference	1.13 (0.88,1.44)	1.17 (0.93,1.48)	1.20 (0.92,1.55)	1.07 (0.81,1.42)	0.30	1.16 (0.73,1.84)
Model 2	Reference	1.13 (0.88,1.45)	1.17 (0.92,1.48)	1.19 (0.91,1.55)	1.10 (0.83,1.45)	0.27	1.21 (0.75,1.92)
Model 3a	Reference	1.14 (0.89,1.46)	1.18 (0.94,1.50)	1.22 (0.93,1.59)	1.13 (0.85,1.51)	0.19	1.28 (0.80,2.06)
Model 3b	Reference	1.17 (0.92,1.51)	1.17 (0.92,1.48)	1.20 (0.92,1.56)	1.11 (0.84,1.47)	0.27	1.20 (0.75,1.92)
**CHD (n=3,691)**							
Mean (min, max)	0.24 (0.00, 0.40)	0.44 (0.41, 0.46)	0.54 (0.46, 0.57)	0.61 (0.57, 0.63)	0.75 (0.63, 1.00)		
Cases	79	97	121	85	65		
Model 1	Reference	1.19 (0.88,1.61)	1.19 (0.89,1.59)	1.34 (0.97,1.84)	1.02 (0.72,1.46)	0.37	1.08 (0.61,1.92)
Model 2	Reference	1.22 (0.90,1.65)	1.19 (0.89,1.59)	1.35 (0.98,1.87)	1.05 (0.74,1.50)	0.33	1.13 (0.64,2.01)
Model 3a	Reference	1.24 (0.91,1.68)	1.20 (0.89,1.61)	1.38 (1.00,1.91)	1.03 (0.72,1.48)	0.34	1.13 (0.63,2.03)
Model 3b	Reference	1.27 (0.93,1.72)	1.18 (0.88,1.58)	1.34 (0.97,1.85)	1.04 (0.73,1.48)	0.40	1.08 (0.61,1.92)
**Stroke (n=3,691)**							
Mean (min, max)	0.24 (0.00, 0.40)	0.44 (0.41, 0.46)	0.54 (0.46, 0.57)	0.61 (0.57, 0.63)	0.75 (0.63, 1.00)		
Cases	48	40	63	27	34		
Model 1	Reference	0.81 (0.52,1.24)	0.98 (0.66,1.44)	0.71 (0.43,1.15)	0.98 (0.61,1.57)	0.66	0.99 (0.44,2.23)

Model 2	Reference	0.80 (0.52,1.24)	0.97 (0.66,1.43)	0.70 (0.43,1.14)	1.01 (0.62,1.62)	0.68	1.03 (0.45,2.35)
Model 3a	Reference	0.80 (0.52,1.23)	0.97 (0.66,1.44)	0.71 (0.44,1.17)	1.14 (0.70,1.86)	0.93	1.18 (0.51,2.74)
Model 3b	Reference	0.82 (0.53,1.27)	0.97 (0.65,1.43)	0.72 (0.44,1.17)	1.03 (0.64,1.66)	0.74	1.05 (0.46,2.40)

*Hazard ratios for 20g/day increase in protein intake measures or 5% increase in energy from protein intake measures.

Model 1 is adjusted for age (years), sex, race/ethnicity (white, Chinese, Black, Hispanic), field center, and total energy intake(kcal) in Model 1.

Model 2 is adjusted all variables in Model 1 plus education (<high school, high school, > high school), income (<$20000, $20000-$49999, >$49999), smoking status (current, former, never) and pack-years of cigarette smoking, dietary supplement use 1 per week or more (yes/no), alcohol use (g/day), physical activity (moderate and vigorous physical activity total, metabolic equivalents per min/wk), prevalent diabetes (yes/no), and body max index (kg/m2).

Model 3a is adjusted all variables in Model 2 plus for intakes of total dietary fiber (g/day), trans fats (% of energy), saturated fatty acids (% of energy), poly-unsaturated fatty acids (% of energy), mono-unsaturated fatty acids (% of energy), vitamin E (IU/day).

Model 3b is adjusted all variables in Model 2 plus for systolic blood pressure (mmHg), diastolic blood pressure (mmHg), triglycerides (mg/dl), HDL cholesterol (mg/dl), LDL cholesterol (mg/dl), C-reactive protein (mg/l), and interleukin-6 (pg/ml).

Table S17. Hazard Ratios (95% CIs) of Incident CVD by Quintile and Continuous Protein Intake Measures Stratified by Race/Ethnicity in MESA (n=5879)

	White (441/2366)	Black/African American (260/1525)	Hispanic (241/1297)	Chinese (103/691)	All (1045/5879)
Total protein (g/day)					
HR per 20g/day increment	1.17 (1.02, 1.35)	0.91 (0.77, 1.08)	1.02 (0.83, 1.25)	0.89 (0.60, 1.31)	1.04 (0.95,1.14)
Animal protein (g/day)					
HR per 20g/day increment	1.15 (1.02, 1.31)	0.96 (0.82, 1.13)	0.96 (0.80, 1.15)	0.82 (0.58, 1.16)	1.04 (0.96,1.13)
Plant protein (g/day)					
HR per 20g/day increment	0.95 (0.68, 1.31)	0.78 (0.53, 1.15)	1.30 (0.89, 1.88)	1.54 (0.78, 3.05)	0.99 (0.81,1.20)
% of energy from Total protein					
HR per 5% increment	1.21 (1.03, 1.41)	0.91 (0.74, 1.12)	1.04 (0.83, 1.30)	0.97 (0.70, 1.35)	1.07 (0.97,1.19)
% of energy from Animal protein					
HR per 5% increment	1.17 (1.02, 1.35)	0.97 (0.80, 1.17)	1.01 (0.82, 1.23)	0.91 (0.68, 1.21)	1.07 (0.97,1.17)
% of energy from Plant protein					
HR per 5% increment	0.97 (0.67, 1.39)	0.77 (0.49, 1.21)	1.10 (0.71, 1.70)	1.47 (0.79, 2.71)	0.97 (0.78,1.21)

*All interactions were not statistically significant based on FDR corrected p-values.

Table S18. Hazard Ratios (95% CIs) of Incident Hypertension by Quintile and Continuous Protein Intake Measures Stratified by Sex in MESA (n=5879)

	Women (447/3081)	Men (598/2798)	All (1045/5879)
Total protein (g/day)			
HR per 20g/day increment	1.18 (1.01, 1.38)	0.98 (0.87, 1.10)	1.04 (0.95,1.14)
Animal protein (g/day)			
HR per 20g/day increment	1.14 (0.99, 1.32)	0.99 (0.89, 1.10)	1.04 (0.96,1.13)
Plant protein (g/day)			
HR per 20g/day increment	1.03 (0.74, 1.43)	0.97 (0.76, 1.24)	0.99 (0.81,1.20)
% of energy from Total protein			
HR per 5% increment	1.19 (1.02, 1.38)	0.98 (0.85, 1.13)	1.07 (0.97,1.19)
% of energy from Animal protein			
HR per 5% increment	1.16 (1.02, 1.33)	0.98 (0.86, 1.12)	1.07 (0.97,1.17)
% of energy from Plant protein			
HR per 5% increment	0.93 (0.67, 1.30)	1.02 (0.76, 1.36)	0.97 (0.78,1.21)

*All interactions were not statistically significant based on FDR corrected p-values.

Table S19. Hazard Ratios (95% CIs) of Incident Hypertension by Quintile and Continuous Protein Intake Measures Stratified by Age in MESA (n=5879)

	<65y (379/3281)	≥ 65y (666/2598)	All (1045/5879)
Total protein (g/day)			
HR per 20g/day increment	1.01 (0.88, 1.16)	1.06 (0.94, 1.20)	1.04 (0.95,1.14)
Animal protein (g/day)			
HR per 20g/day increment	1.01 (0.89, 1.15)	1.06 (0.95, 1.18)	1.04 (0.96,1.13)
Plant protein (g/day)			
HR per 20g/day increment	0.98 (0.71, 1.37)	0.96 (0.76, 1.23)	0.99 (0.81,1.20)
% of energy from Total protein			
HR per 5% increment	0.98 (0.83, 1.16)	1.12 (0.98, 1.28)	1.07 (0.97,1.19)
% of energy from Animal protein			
HR per 5% increment	1.00 (0.86, 1.16)	1.10 (0.98, 1.24)	1.07 (0.97,1.17)
% of energy from Plant protein			
HR per 5% increment	0.91 (0.63, 1.33)	0.99 (0.76, 1.30)	0.97 (0.78,1.21)

*All interactions were not statistically significant based on FDR corrected p-values.

Table S20. Hazard Ratios (95% CIs) of Incident Hypertension by Quintile and Continuous Protein Intake Measures Stratified by Education in MESA (n=5879)

	High school graduate or less (437/ 2097)	Some college and above (608/ 3782)	All (1045/5879)
Total protein (g/day)			
HR per 20g/day increment	1.04 (0.90, 1.20)	1.05 (0.94, 1.19)	1.04 (0.95,1.14)
Animal protein (g/day)			
HR per 20g/day increment	1.01 (0.88, 1.15)	1.06 (0.95, 1.19)	1.04 (0.96,1.13)
Plant protein (g/day)			
HR per 20g/day increment	1.14 (0.85, 1.54)	0.92 (0.71, 1.19)	0.99 (0.81,1.20)
% of energy from Total protein			
HR per 5% increment	1.09 (0.93, 1.28)	1.08 (0.94, 1.24)	1.07 (0.97,1.19)
% of energy from Animal protein			
HR per 5% increment	1.04 (0.90, 1.20)	1.09 (0.97, 1.23)	1.07 (0.97,1.17)
% of energy from Plant protein			
HR per 5% increment	1.16 (0.83, 1.62)	0.88 (0.65, 1.18)	0.97 (0.78,1.21)

*All interactions were not statistically significant based on FDR corrected p-values.

Table S21. Hazard Ratios (95% CIs) of Incident Hypertension by Quintile and Continuous Protein Intake Measures Stratified by Income in MESA (n=5879)

	<$20000 (305/1359)	$20000-$49999 (419/2188)	≥50000 (321/2332)	All (1045/5879)
Total protein (g/day)				
HR per 20g/day increment	1.11 (0.94, 1.31)	0.96 (0.83, 1.12)	1.08 (0.92, 1.27)	1.04 (0.95,1.14)
Animal protein (g/day)				
HR per 20g/day increment	1.06 (0.91, 1.23)	0.97 (0.85, 1.12)	1.08 (0.93, 1.25)	1.04 (0.96,1.13)
Plant protein (g/day)				
HR per 20g/day increment	1.19 (0.83, 1.69)	0.99 (0.72, 1.36)	0.92 (0.65, 1.32)	0.99 (0.81,1.20)
% of energy from Total protein				
HR per 5% increment	1.22 (1.02, 1.47)	0.99 (0.84, 1.16)	1.05 (0.87, 1.27)	1.07 (0.97,1.19)
% of energy from Animal protein				
HR per 5% increment	1.13 (0.96, 1.33)	1.00 (0.86, 1.16)	1.07 (0.90, 1.27)	1.07 (0.97,1.17)
% of energy from Plant protein				
HR per 5% increment	1.22 (0.83, 1.79)	0.97 (0.68,1.39)	0.87 (0.58,1.32)	0.97 (0.78,1.21)

*All interactions were not statistically significant based on FDR corrected p-values.

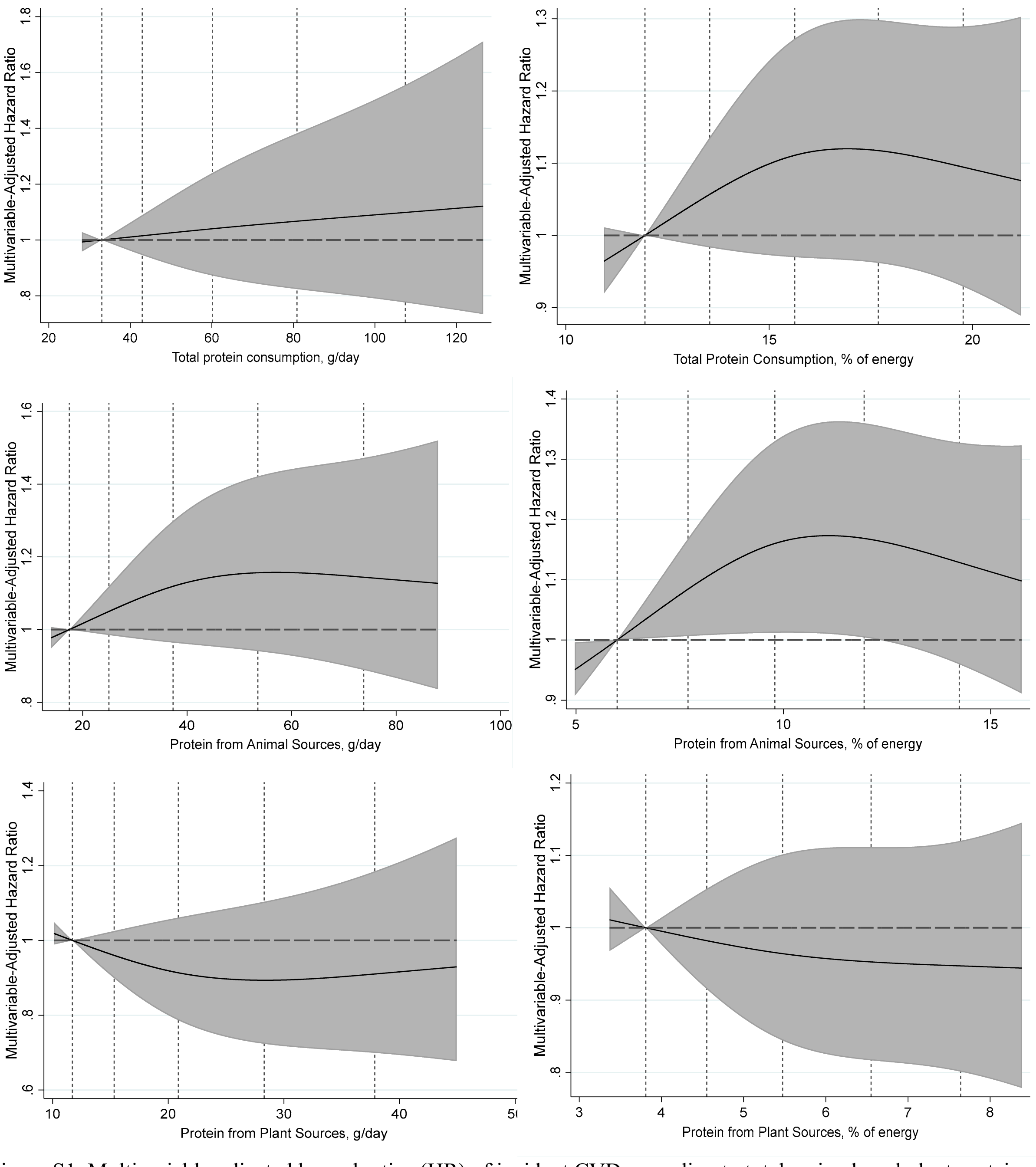

Figure S1. Multivariable-adjusted hazard ratios (HR) of incident CVD according to total, animal, and plant protein intake (g/day and % energy), evaluated using restricted cubic splines. Solid lines and shaded area represent the HR estimate and 95% confidence interval, respectively. The dotted vertical lines represent the 10th, 25th, 50th, 75th, and 90th percentiles. The top and bottom 5% of participants were omitted for visualization. P values for non-linearity: 0.87 (total protein consumption, g/day), 0.14 (animal protein consumption, g/day), 0.21 (plant protein consumption, g/day), 0.22 (total protein consumption, % energy), 0.05 (animal protein consumption, % energy), and 0.77 (plant protein consumption, % energy). The multivariable models were adjusted for age, sex, race/ethnicity, field center, total energy intake, education, income, smoking status, pack-years of cigarette smoking, dietary supplement use, alcohol use, physical activity, prevalent diabetes, and body mass index.

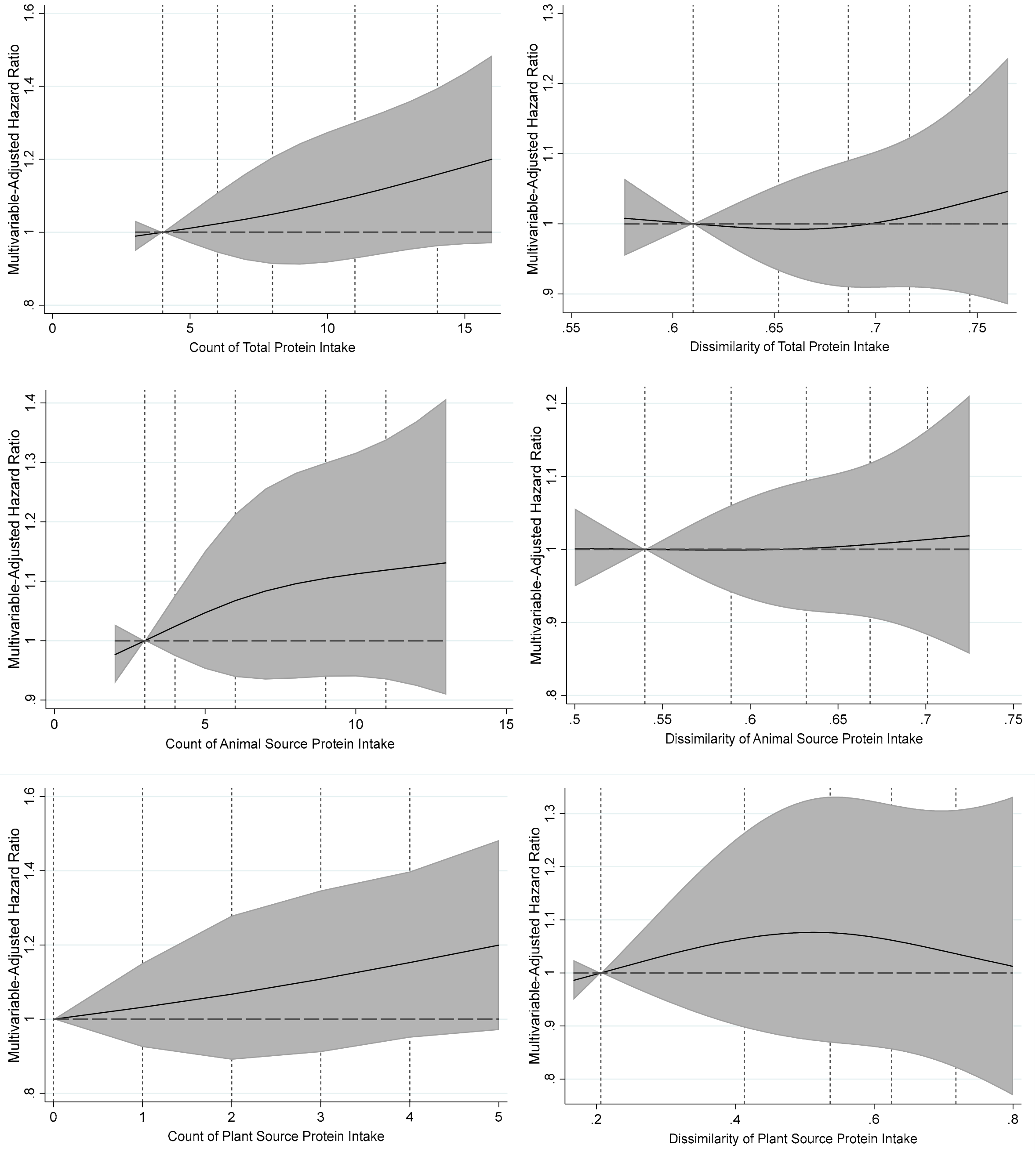

Figure S2. Multivariable-adjusted hazard ratios (HR) of incident CVD according to diversity of protein sources, evaluated using restricted cubic splines. Solid lines and shaded area represent the HR estimate and 95% confidence interval, respectively. The dotted vertical lines represent the 10th, 25th, 50th, 75th, and 90th percentiles. The top and bottom 5% of participants were omitted for visualization. P values for non-linearity: 0.90 (count of total protein intake), 0.58 (count of animal source protein intake), 0.95 (count of plant source protein intake), 0.59 (dissimilarity of total protein intake), 0.89 (dissimilarity of animal source protein intake), and 0.47 (dissimilarity of plant source protein intake). The multivariable models were adjusted for age, sex, race/ethnicity, field center, total energy intake, education, income, smoking status, pack-years of cigarette smoking, dietary supplement use, alcohol use, physical activity, prevalent diabetes, and body mass index.

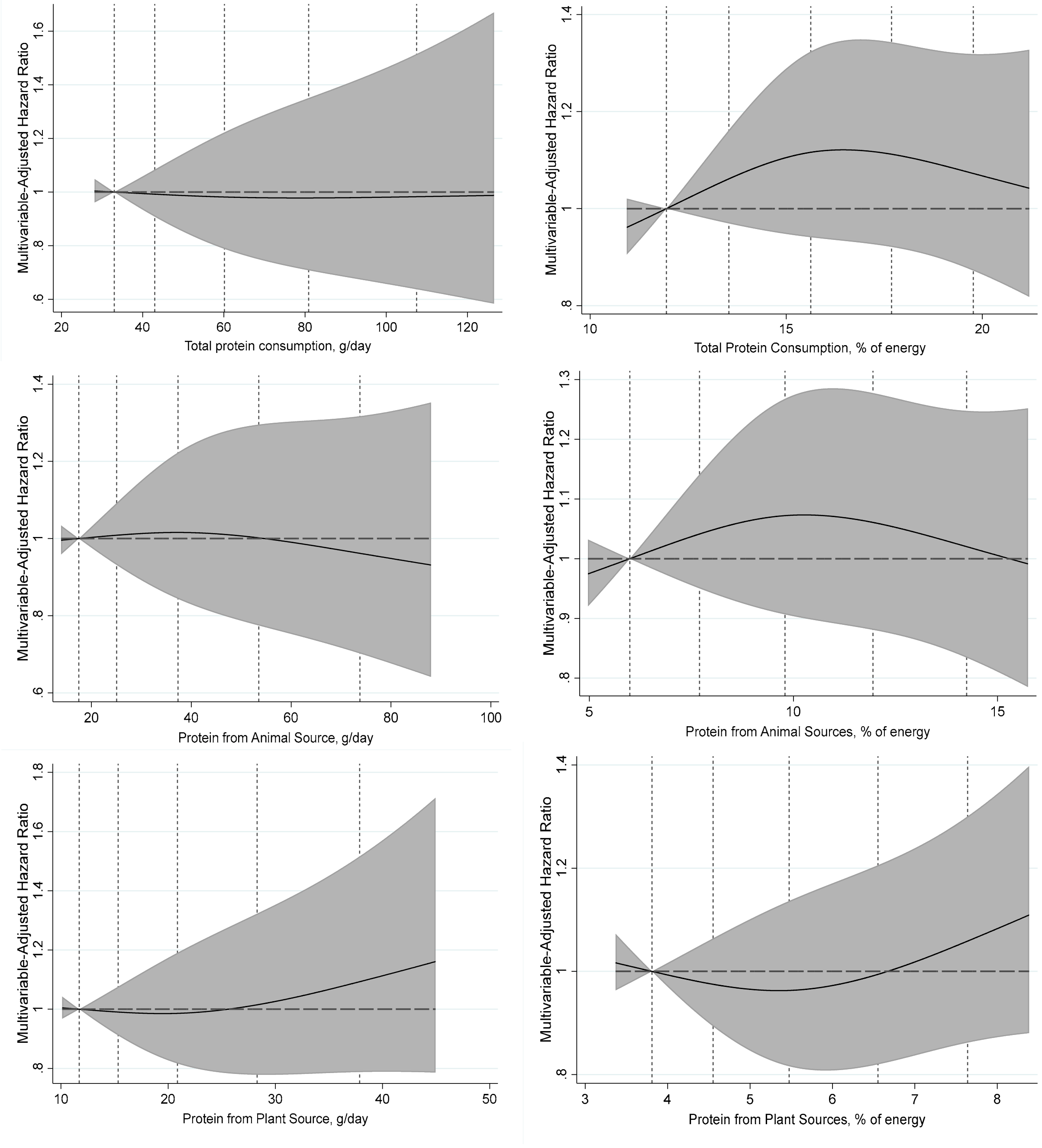

Figure S3. Multivariable-adjusted hazard ratios (HR) of incident CHD according to total, animal, and plant protein intake (g/day and % energy), evaluated using restricted cubic splines. Solid lines and shaded area represent the HR estimate and 95% confidence interval, respectively. The dotted vertical lines represent the 10th, 25th, 50th, 75th, and 90th percentiles. The top and bottom 5% of participants were omitted for visualization. P values for non-linearity: 0.88 (total protein consumption, g/day), 0.57 (animal protein consumption, g/day), 0.44 (plant protein consumption, g/day), 0.24 (total protein consumption, % energy), 0.31 (animal protein consumption, % energy), and 0.26 (plant protein consumption, % energy). The multivariable models were adjusted for age, sex, race/ethnicity, field center, total energy intake, education, income, smoking status, pack-years of cigarette smoking, dietary supplement use, alcohol use, physical activity, prevalent diabetes, and body mass index.

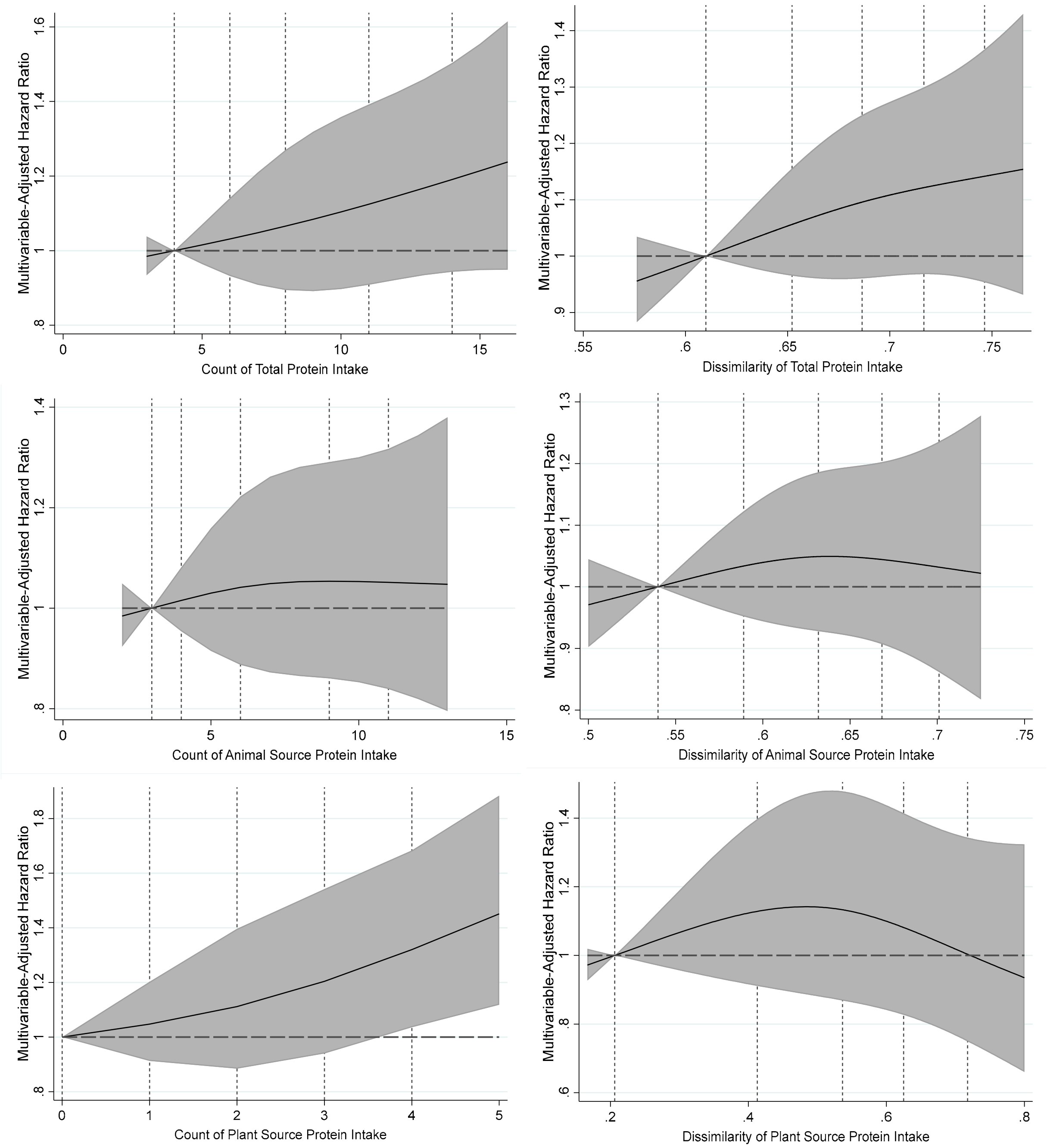

Figure S4. Multivariable-adjusted hazard ratios (HR) of incident CHD according to diversity of protein sources, evaluated using restricted cubic splines. Solid lines and shaded area represent the HR estimate and 95% confidence interval, respectively. The dotted vertical lines represent the 10th, 25th, 50th, 75th, and 90th percentiles. The top and bottom 5% of participants were omitted for visualization. P values for non-linearity: 0.99 (count of total protein intake), 0.68 (count of animal source protein intake), 0.68 (count of plant source protein intake), 0.71 (dissimilarity of total protein intake), 0.54 (dissimilarity of animal source protein intake), and 0.18 (dissimilarity of plant source protein intake). The multivariable models were adjusted for age, sex, race/ethnicity, field center, total energy intake, education, income, smoking status, pack-years of cigarette smoking, dietary supplement use, alcohol use, physical activity, prevalent diabetes, and body mass index.

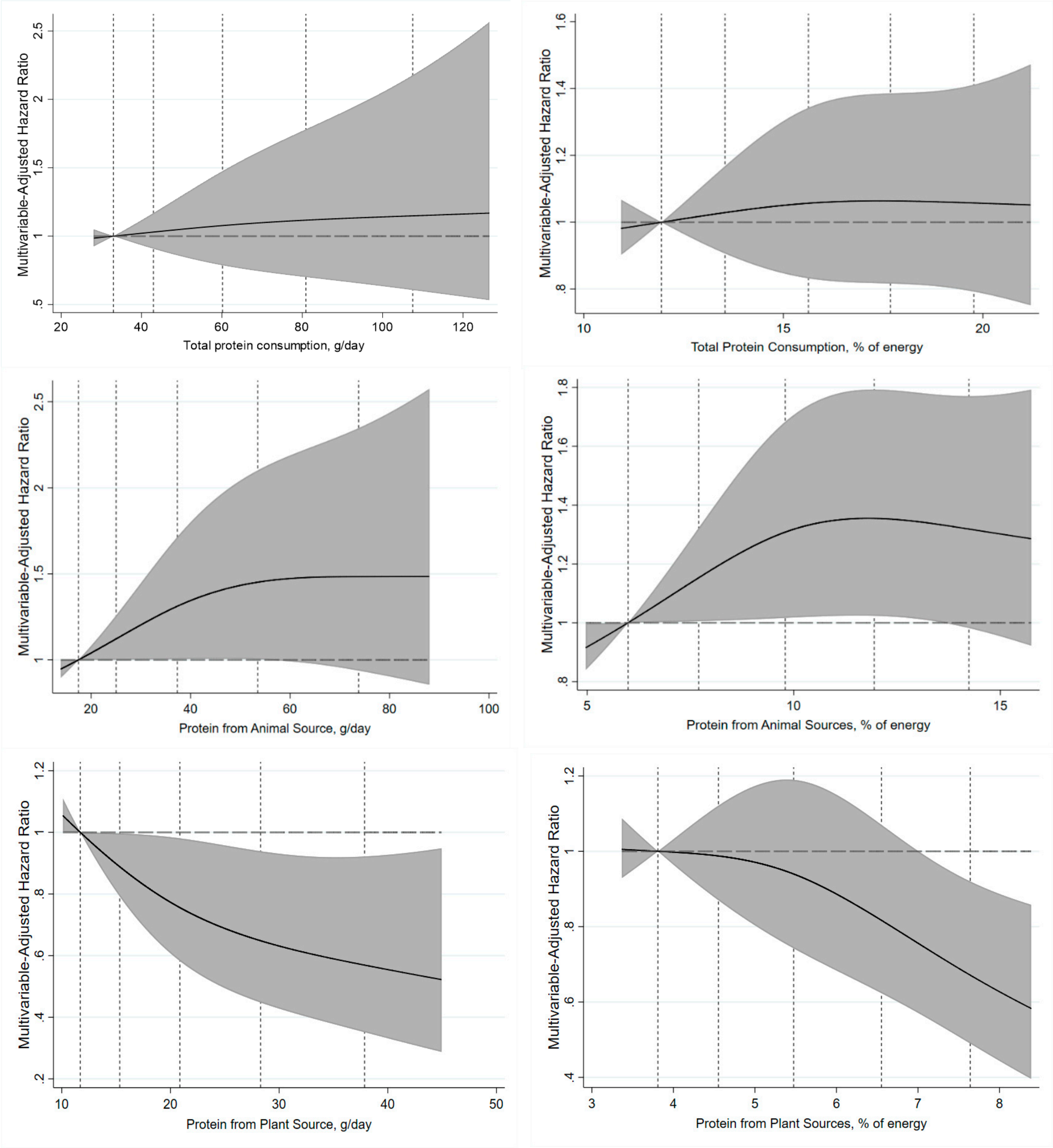

Figure S5. Multivariable-adjusted hazard ratios (HR) of incident stroke according to total, animal, and plant protein intake (g/day and % energy), evaluated using restricted cubic splines. Solid lines and shaded area represent the HR estimate and 95% confidence interval, respectively. The dotted vertical lines represent the 10th, 25th, 50th, 75th, and 90th percentiles. The top and bottom 5% of participants were omitted for visualization. P values for non-linearity: 0.74 (total protein consumption, g/day), 0.11 (animal protein consumption, g/day), 0.33 (plant protein consumption, g/day), 0.73 (total protein consumption, % energy), 0.10 (animal protein consumption, % energy), and 0.23 (plant protein consumption, % energy). The multivariable models were adjusted for age, sex, race/ethnicity, field center, total energy intake, education, income, smoking status, pack-years of cigarette smoking, dietary supplement use, alcohol use, physical activity, prevalent diabetes, and body mass index.

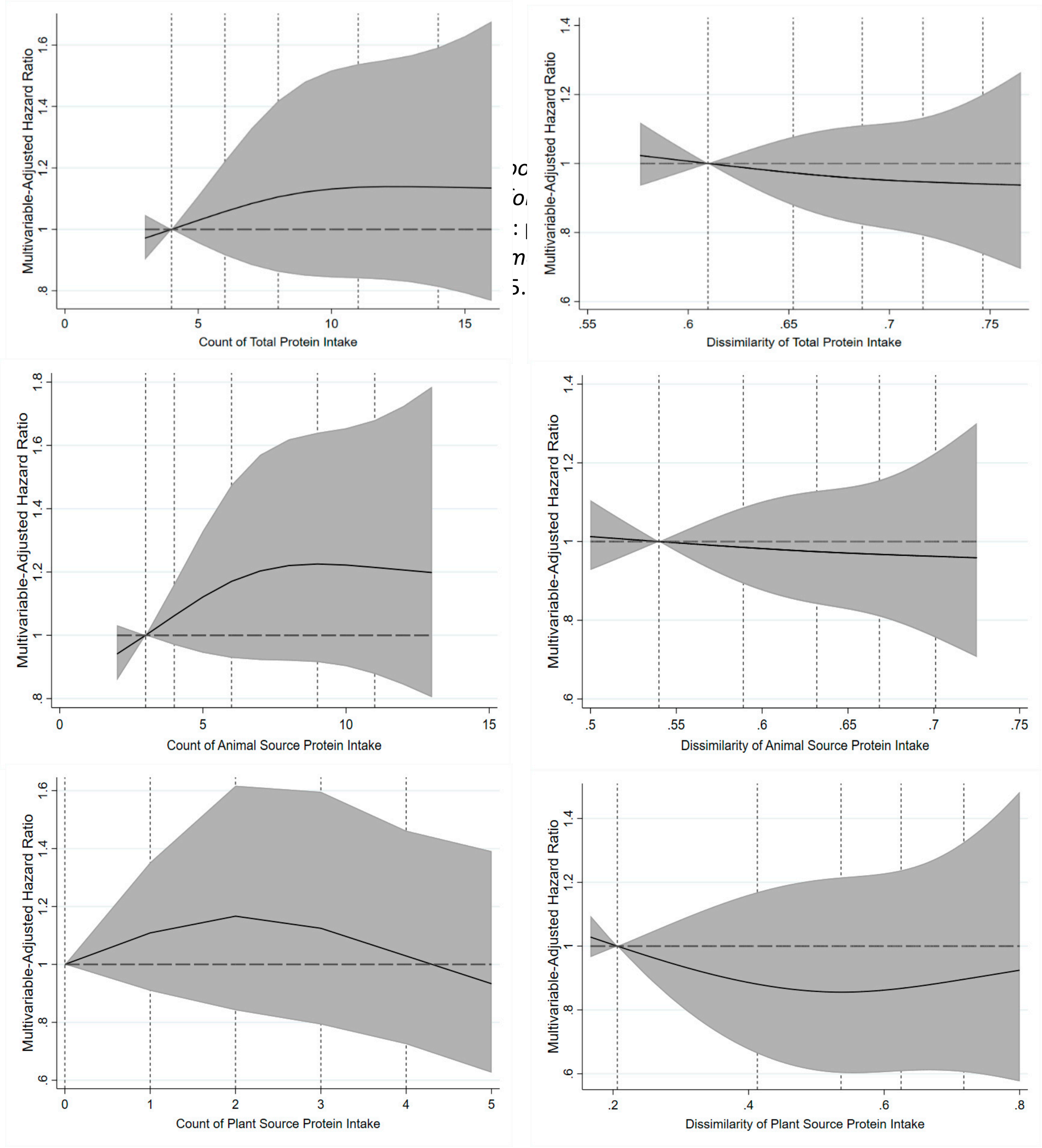

Figure S6. Multivariable-adjusted hazard ratios (HR) of incident stroke according to diversity of protein sources, evaluated using restricted cubic splines. Solid lines and shaded area represent the HR estimate and 95% confidence interval, respectively. The dotted vertical lines represent the 10th, 25th, 50th, 75th, and 90th percentiles. The top and bottom 5% of participants were omitted for visualization. P values for non-linearity: 0.56 (count of total protein intake), 0.30 (count of animal source protein intake), 0.18 (count of plant source protein intake), 0.90 (dissimilarity of total protein intake), 0.98 (dissimilarity of animal source protein intake), and 0.53 (dissimilarity of plant source protein intake). The multivariable models were adjusted for age, sex, race/ethnicity, field center, total energy intake, education, income, smoking status, pack-years of cigarette smoking, dietary supplement use, alcohol use, physical activity, prevalent diabetes, and body mass index.

References

1. Pounis, G., et al. *Consumption of healthy foods at different content of antioxidant vitamins and phytochemicals and metabolic risk factors for cardiovascular disease in men and women of the Moli–sani study.* European Journal of Clinical Nutrition, 2013. 67.2(207-213).

2. de Oliveira Otto, M.C., et al., *Everything in moderation-dietary diversity and quality, central obesity and risk of diabetes.* PLoS One, 2015. 10(10): p. e0141341.
